# Supplementary material for: Awareness, treatment, and control of hypertension in adults aged 45 years and over and their spouses in India: A nationally representative cross-sectional study
Source: PLoS Med. 2021 Aug 24;18(8):e1003740. doi: 10.1371/journal.pmed.1003740 (PMC8425529; doi:10.1371/journal.pmed.1003740)
Supplement: S1 Checklist — (DOC) [file pmed.1003740.s001.doc]

**S1 Checklist.** STROBE checklist of items that should be included in reports of **cross-sectional studies**

|  | Item No | Recommendation | Section, paragraph Response |
| --- | --- | --- | --- |
| **Title and abstract** | 1 | (*a*) Indicate the study’s design with a commonly used term in the title or the abstract | Title |
|  |  | (*b*) Provide in the abstract an informative and balanced summary of what was done and what was found | Abstract: Methods & findings. Author summary: Why was this study was done? What did the researchers do and find? |
| Introduction | | |  |
| Background/ rationale | 2 | Explain the scientific background and rationale for the investigation being reported | Abstract: Background. Author summary: Why was this study done? Introduction |
| Objectives | 3 | State specific objectives, including any prespecified hypotheses | Abstract: Background. Introduction: final paragraph. |
| Methods | | |  |
| Study design | 4 | Present key elements of study design early in the paper | Abstract: Methods and findings. Introduction: final paragraph. Methods: Study design & participants |
| Setting | 5 | Describe the setting, locations, and relevant dates, including periods of recruitment, exposure, follow-up, and data collection | Methods: Study design and participants |
| Participants | 6 | (*a*) Give the eligibility criteria, and the sources and methods of selection of participants | Methods: Study design and participants. Results: paragraph 1. S1 Text |
| Variables | 7 | Clearly define all outcomes, exposures, predictors, potential confounders, and effect modifiers. Give diagnostic criteria, if applicable | Methods: Measures, Statistical analysis. S2 Text. S3 Text. |
| Data sources/ measurement | 8* | For each variable of interest, give sources of data and details of methods of assessment (measurement). Describe comparability of assessment methods if there is more than one group | Methods: Study design and participants, Measures |
| Bias | 9 | Describe any efforts to address potential sources of bias | Methods: Study design and participants, Statistical analysis (final paragraph). Results: first paragraph |
| Study size | 10 | Explain how the study size was arrived at | Methods: Study design and participants. Results: first paragraph. Figure 1 |
| Quantitative variables | 11 | Explain how quantitative variables were handled in the analyses. If applicable, describe which groupings were chosen and why | Methods: Statistical analysis. Table 1, notes |
| Statistical methods | 12 | (*a*) Describe all statistical methods, including those used to control for confounding | Methods: Statistical analysis. S4 Text |
|  |  | (*b*) Describe any methods used to examine subgroups and interactions | Methods: Statistical analysis |
|  |  | (*c*) Explain how missing data were addressed | Figure 1. Methods: final paragraph 7. Results: first paragraph. S1-S3 Table |
|  |  | (*d*) If applicable, describe analytical methods taking account of sampling strategy | Methods: final paragraph |
|  |  | (*e*) Describe any sensitivity analyses | S1-S3 Table. S6 Table. S9 Table |
| Results | | |  |
| Participants | 13 | (a) Report numbers of individuals at each stage of study—eg numbers potentially eligible, examined for eligibility, confirmed eligible, included in the study, completing follow-up, and analysed | Fig 1 |
|  |  | (b) Give reasons for non-participation at each stage | Fig 1 |
|  |  | (c) Consider use of a flow diagram | Fig 1 |
| Descriptive data | 14* | (a) Give characteristics of study participants (eg demographic, clinical, social) and information on exposures and potential confounders | Table 1 |
|  |  | (b) Indicate number of participants with missing data for each variable of interest | S2 Table |
| Outcome data | 15* | Report numbers of outcome events or summary measures | Table 1 and Table 3 |
| Main results | 16 | (*a*) Give unadjusted estimates and, if applicable, confounder-adjusted estimates and their precision (eg, 95% confidence interval). Make clear which confounders were adjusted for and why they were included | S6 Table and S9 Table. Table 1 and Table 3. Fig 4 and S11 Table |
|  |  | (*b*) Report category boundaries when continuous variables were categorized | Tables 1 |
|  |  | (*c*) If relevant, consider translating estimates of relative risk into absolute risk for a meaningful time period | Not Applicable |
| Other analyses | 17 | Report other analyses done—eg analyses of subgroups and interactions, and sensitivity analyses | S7 Table and S10 Table |
| Discussion | | |  |
| Key results | 18 | Summarise key results with reference to study objectives | Abstract: Methods and findings, Conclusions. Discussion: paragraphs 1-5) |
| Limitations | 19 | Discuss limitations of the study, taking into account sources of potential bias or imprecision. Discuss both direction and magnitude of any potential bias | Abstract: Methods and findings. Discussion, paragraphs 9, 10, 11 |
| Interpretation | 20 | Give a cautious overall interpretation of results considering objectives, limitations, multiplicity of analyses, results from similar studies, and other relevant evidence | Discussion |
| Generalis-ability | 21 | Discuss the generalisability (external validity) of the study results | Discussion: final paragraph |
| Other information | | |  |
| Funding | 22 | Give the source of funding and the role of the funders for the present study and, if applicable, for the original study on which the present article is based | Provided during submission process |
