# Supplementary material for: Awareness, treatment, and control of hypertension in adults aged 45 years and over and their spouses in India: A nationally representative cross-sectional study
Source: PLoS Med. 2021 Aug 24;18(8):e1003740. doi: 10.1371/journal.pmed.1003740 (PMC8425529; doi:10.1371/journal.pmed.1003740)
Supplement: S1 Text — (DOCX) [file pmed.1003740.s002.docx]

**S1 Text. Sample design**

Longitudinal Aging Study in India (LASI) is the first-ever longitudinal study on the health, economic and social well-being of older adults (45+) and their spouses in India. LASI adopted a multistage stratified area probability cluster sampling design. In rural areas, there was a three-stage sampling design, while in urban areas, there was a four-stage sampling design. In the first stage, primary sampling units (PSU) (sub-districts or Tehsils/Talukas) were selected using the probability proportional to sample size (PPS) sampling. In the second stage, secondary sampling units (SSU), which were villages in rural areas and wards in urban areas, were selected from the selected PSUs. In the third stage, a fixed number of households were randomly selected from each selected village in the rural areas. In urban areas, Census Enumeration Blocks (CEB) were randomly selected and then households were randomly sampled within each selected CEB.

The first level of stratification was geographic regions, with a group of districts forming a contiguous region (adopted from National Sample Survey Office regional classifications). Within each of these regions, all the PSUs (sub-districts) were explicitly stratified using total number of households in a sub-district, level of female literacy, the proportion of Scheduled caste and Scheduled tribe population, and the proportion of males engaged in the non-agricultural sector. Before the selection of PSUs from each stratum using the PPS sampling technique, the sub-districts were arranged according to the level of female literacy (implicit stratification) to ensure a representative sample of sub-districts with varying levels of social development.

Households (HH) were randomly selected from the house listing for each of the SSUs (rural villages and urban CEBs), in which each of the household was serially numbered. HHs were selected in two phases. For each CEB, 31 HHs were selected from all HHs listed in the CEB (irrespective of their eligibility criteria) using the systematic random sampling method. Then, 4 additional HHs were selected from all HHs that had at least one member age 65 or above (after excluding the HHs already selected in stratum). A similar approach was used for rural areas, with 28 and 4 HHs, respectively, within each village.

In addition, the survey also included four metropolitan cities as part of a national urban sample. In addition to Delhi (state), which represents the north region, the cities of Kolkata, Mumbai, and Chennai were selected to represent the east, west, and south regions respectively. The goal was to obtain representative samples for each of these four metropolitan cities to generate city-specific indicators and to contribute to regional and national urban indicators. Multistage stratified area probability cluster sampling design was also adopted for sample selection in metro cities. In first stage, city ward PSUs were randomly selected within each of the four metro cities using PPS sampling. In second stage, one CEB from each selected ward was randomly selected. In the third stage, HHs from each CEB were selected using systematic random sampling.

The survey team spent 5 days, on average, in a PSU. At least three visits were made to each HH to complete the interview. The household response rate was 95.8%, while the individual response rate was 87.3%. Further details of the sampling design are given in the LASI report.^1^

^1^ IIPS, NPHCE, MOHFW, Harvard T. H. Chan School of Public Health (HSPH), & University of Southern California (USC). Longitudinal Ageing Study in India Wave 1, 2017-18, Report. Mumbai: International Institute for Population Sciences, 2020.
