# Supplementary material for: Awareness, treatment, and control of hypertension in adults aged 45 years and over and their spouses in India: A nationally representative cross-sectional study
Source: PLoS Med. 2021 Aug 24;18(8):e1003740. doi: 10.1371/journal.pmed.1003740 (PMC8425529; doi:10.1371/journal.pmed.1003740)
Supplement: S2 Text — (DOCX) [file pmed.1003740.s003.docx]

**S2 Text. Outcome definitions**

| **Outcome** | **Definition** |
| --- | --- |
| Hypertension | 1 IF a) SBP ≥140 mm Hg [BM017 ≥ 140] OR DBP ≥ 90 mm Hg [BM018 ≥ 90],  OR b) reported ever told have hypertension/ high BP [HT002=1] AND reported currently taking medication to control BP [HT002c=1] OR reported under salt or diet restriction to control BP [HT002d=1],  0 otherwise. |
| Awareness | 1 IF Hypertension=1 AND reported ever told have hypertension/ high BP [HT002=1],  0 IF Hypertension=1 AND reported never told have hypertension/ high BP [HT002=0],  missing IF Hypertension=0. |
| Treatment | 1 IF Hypertension=1 AND a) reported currently taking medication to control BP [HT002c=1] OR b) reported under salt or diet restriction to control BP [HT002d=1],  0 IF Hypertension=1 AND reported not currently taking medication to control BP [HT002c=0] AND reported not under salt or diet restriction to control BP [HT002d=0],  missing IF Hypertension=0. |
| Control | 1 IF Hypertension=1 AND SBP<140 [BM017<140] AND DBP<90 [BM018 <90],  0 IF Hypertension=1 AND SBP ≥140 [BM017 ≥140] AND DBP ≥90 [BM018 ≥90],  missing IF Hypertension=0. |

| **Question Number** | **Question** | | | | **Response** |
| --- | --- | --- | --- | --- | --- |
| HT002 | Has any health professional ever told you that Hypertension or higher blood pressure | | | | 1.Yes  2. No |
| HT002c | [if HT002=1] In order to control your blood pressure or hypertension, are you currently taking any medication? | | | | 1.Yes  2. No |
| HT002d | [if HT002 =1] In order to control your blood pressure, are you under salt or any diet restrictions? | | | | 1.Yes  2. No |
|  | Blood pressure measurement | | | |  |
| BM005 | When the device is in the correct position and the R is relaxed, press the button to Start. Measure blood pressure and pulse three times with one minute gap between each of the measurements. No need to remove the cuffs and the device between the measurements. Record measurements in CAPI. Enter 993 in systolic, diastolic and pulse reading if an unresolvable equipment problem occurs. If the average systolic reading obtained is greater than 180 and average diastolic reading is greater than 110 or either of it, fill the referral letter and give to respondent and stop the test immediately. | | | | |
| **Measurement** | **Times of reading** | **Systolic reading** | **Diastolic reading** | **Pulse** | |
| **1** | BM005.  __ __:__ __ am/pm | BM006.  ___ mmHg | BM007.  ___ mmHg | \| BM008.___ \| Beats/min \| \| --- \| --- \| | |
| **2** | BM009.  __ __:__ __ am/pm | BM010.  ___ mmHg | BM011.  ___mmHg | \| BM012.___ \| Beats/min \| \| --- \| --- \| | |
| **3** | BM013.  __ __:__ __ am/pm | BM014.  ___ mmHg | BM015.  ___mmHg | \| BM016.___ \| Beats/min \| \| --- \| --- \| | |
| **4** | **Average of last 2 readings** | **BM017.**  **___mmHg** | **BM018. ___mmHg** | \| **BM019. __** \| **Beats/min** \| \| --- \| --- \| | |

| **Statistic** | **Definition** | **Explanation** |
| --- | --- | --- |
| Hypertension prevalence | $\frac{N_{HTN}}{N}\times100$ | $N_{HTN}$= weighted number of participants in analysis sample with Hypertension=1,  N = weighted number of participants in analysis sample |
| Awareness rate | $\frac{N_{A}}{N_{HTN}}\times100$ | $N_{A}$= weighted number of participants in analysis sample with Awareness=1 |
| Treatment rate | $\frac{N_{T}}{N_{HTN}}\times100$ | $N_{T}$= weighted number of participants in analysis sample with Treatment=1 |
| Control rate | $\frac{N_{C}}{N_{HTN}}\times100$ | $N_{C}$= weighted number of participants in analysis sample with Treatment=1 |

Brackets give respective questions and responses. See below. BP = blood pressure, SBP = systolic blood pressure, DBP = diastolic blood pressure.
