# Supplementary material for: Awareness, treatment, and control of hypertension in adults aged 45 years and over and their spouses in India: A nationally representative cross-sectional study
Source: PLoS Med. 2021 Aug 24;18(8):e1003740. doi: 10.1371/journal.pmed.1003740 (PMC8425529; doi:10.1371/journal.pmed.1003740)
Supplement: S3 Text — (DOCX) [file pmed.1003740.s004.docx]

**S3 Text. Measurement of monthly per capita consumption expenditure (MPCE)**

Monthly per capita consumption expenditure (MPCE) is a direct measure of a household’s living standard. Official estimates of poverty and inequality in India are derived from MPCE measured in the National Sample Survey (NSS). The LASI measure of MPCE followed the NSS methodology (an abridged version of consumption schedule), although not to the same level of detail. Household food consumption in10 categories of food was reported for a reference period of the last 7 days. The value of food produced by a household for its own consumption and food eaten outside was included. Non-food expenditure was reported for a reference period of the last 30 days for more frequently purchased items and over the last 365 days for seldom-purchased durable goods. We calculated household expenditure for a 30-day period by multiplying reported food consumption by 30/7 and dividing non-food expenditures reported for the last year by 365/30. We excluded from MPCE out-of-pocket expenditure on healthcare and medicines in order to avoid such expenditures, which may well be temporary and not financed from a household’s current income, inflating total household expenditure and giving a false impression of a higher standard of living. Without doing so, there would be a risk of misclassifying households with sickness and medical spending as economically better off than households without such spending. The variables used in derivation of MPCE are similar to that of NSS. After adjusting for the different reference periods and aggregating across categories of expenditure, we divided by household size to get MPCE. In the analysis sample of individuals, we applied sampling weights to get the quintiles of MPCE and used these values to categorize participants into five groups of equal (weighted) size: poorest 20%, 2^nd^ poorest 20%, middle 20%, richer 20% and richest 20%.
