# Supplementary material for: Awareness, treatment, and control of hypertension in adults aged 45 years and over and their spouses in India: A nationally representative cross-sectional study
Source: PLoS Med. 2021 Aug 24;18(8):e1003740. doi: 10.1371/journal.pmed.1003740 (PMC8425529; doi:10.1371/journal.pmed.1003740)
Supplement: S4 Text — (DOCX) [file pmed.1003740.s005.docx]

**S4 Text. Age-sex adjustment**

We adjusted estimates of hypertension prevalence, awareness, treatment, and control for age and sex by using the age-sex composition of the nationally representative full sample as the reference. For example, to obtain age-sex adjusted prevalence of hypertension by state, we estimated a logistic regression of the binary hypertension outcome on a full set of state indicators (fixed effects) and 36 sex-specific age group (<45 years, 45-46, 46-47, 48-49, …, 69-70, 71-75, 76-80, 81-85, and 85+) indicators (fixed effects) with sample weights applied. Then, for each state, we averaged the predicted probability of having hypertension if located in that state over all sample participants, i.e. the average adjusted prediction. This gave an estimate of what hypertension prevalence would be in a state if its age-sex composition was the same as that of the whole sample, which was representative of the national population aged 45+, plus spouses, when sample weights were applied. We obtained analogous estimates for all outcomes (prevalence, awareness, treatment and control), and for the means of these outcomes across MPCE quintile groups and sociodemographic characteristics. In the latter case, the logistic regression from which the averaged predictions of the respective outcome were obtained included MPCE quintle group indicators (or categories of the sociodemographic characteristic) and the 36 age-sex categories fixed effects.

We used indirect standardization to adjust concentration curves and indices for age and sex.^1^ For example, we estimated a logistic regression of the binary hypertension outcome on the 36 sex-specific age group indicators, and used the estimates to predict the probability of hypertension for each participant given their age and sex. We then subtracted this prediction from the hypertension outcome of each participant and added the mean prediction. We then traced the concentration curve of this age-sex adjusted indicator of hypertension status. To estimate the adjusted concentration index, we did not add the mean prediction to the deviation of actual hypertension outcome from the age-sex predicted outcome to ensure that the adjusted outcome was in the range of (-1,1). Since the concentration index used Erreygers (2009) measures absolute inequality, it makes no difference if a constant mean is added to all observations.^2^

^1^Wagstaff A, O'Donnell O, Van Doorslaer E, Lindelow M. Analyzing health equity using household survey data: a guide to techniques and their implementation. World Bank Publications; 2007 Nov
