# Supplementary figures and images for: Awareness, treatment, and control of hypertension in adults aged 45 years and over and their spouses in India: A nationally representative cross-sectional study
Source: PLoS Med. 2021 Aug 24;18(8):e1003740. doi: 10.1371/journal.pmed.1003740 (PMC8425529; doi:10.1371/journal.pmed.1003740)

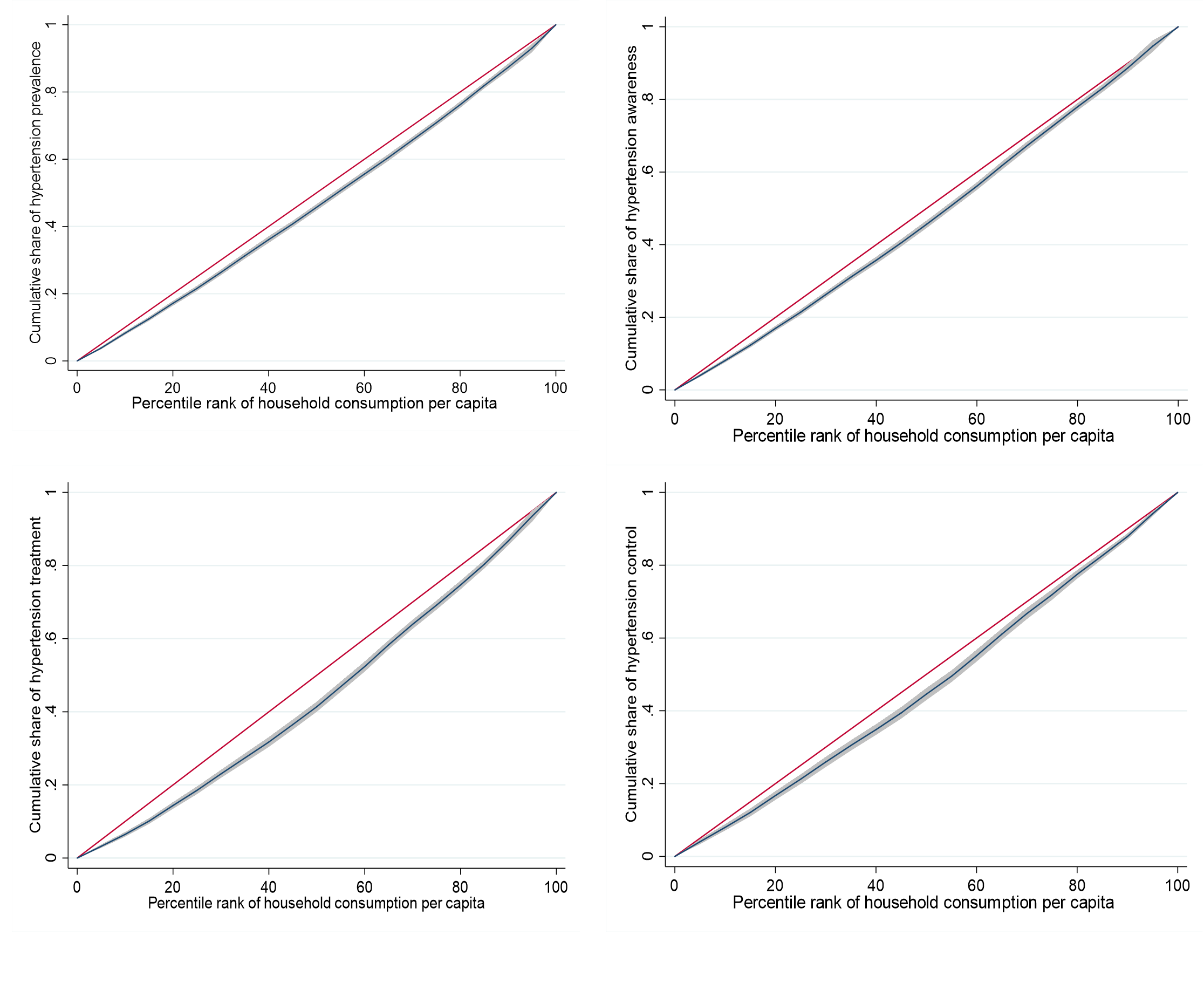

Supplement: S1 Fig — The figure shows concentration curves, which depict relative inequality in an outcome in relation to a measure of socioeconomic status (O’Donnell and colleagues 2008). For example, the curve in the top-left panel traces the cumulative proportion of hypertension cases (y-axis) against the cumulative proportion of participants ranked from the poorest (left) to the richest (right) (x-axis) based on MPCE. The other curves trace the cumulative proportion of participants with hypertension who are aware, treated, and controlled (y-axis) against the cumulative proportion ranked from poorest to richest. Shading around the curves indicates 95% CIs. Each curve lies below the respective 45-degree line, which indicates that there is a disproportionate concentration of hypertension cases among richer participants and that among those with hypertension, ATC are also disproportionately concentrated among the richer participants. ATC, awareness, treatment, and control; MPCE, monthly per capita consumption expenditure. (TIF) [file pmed.1003740.s006.tif]

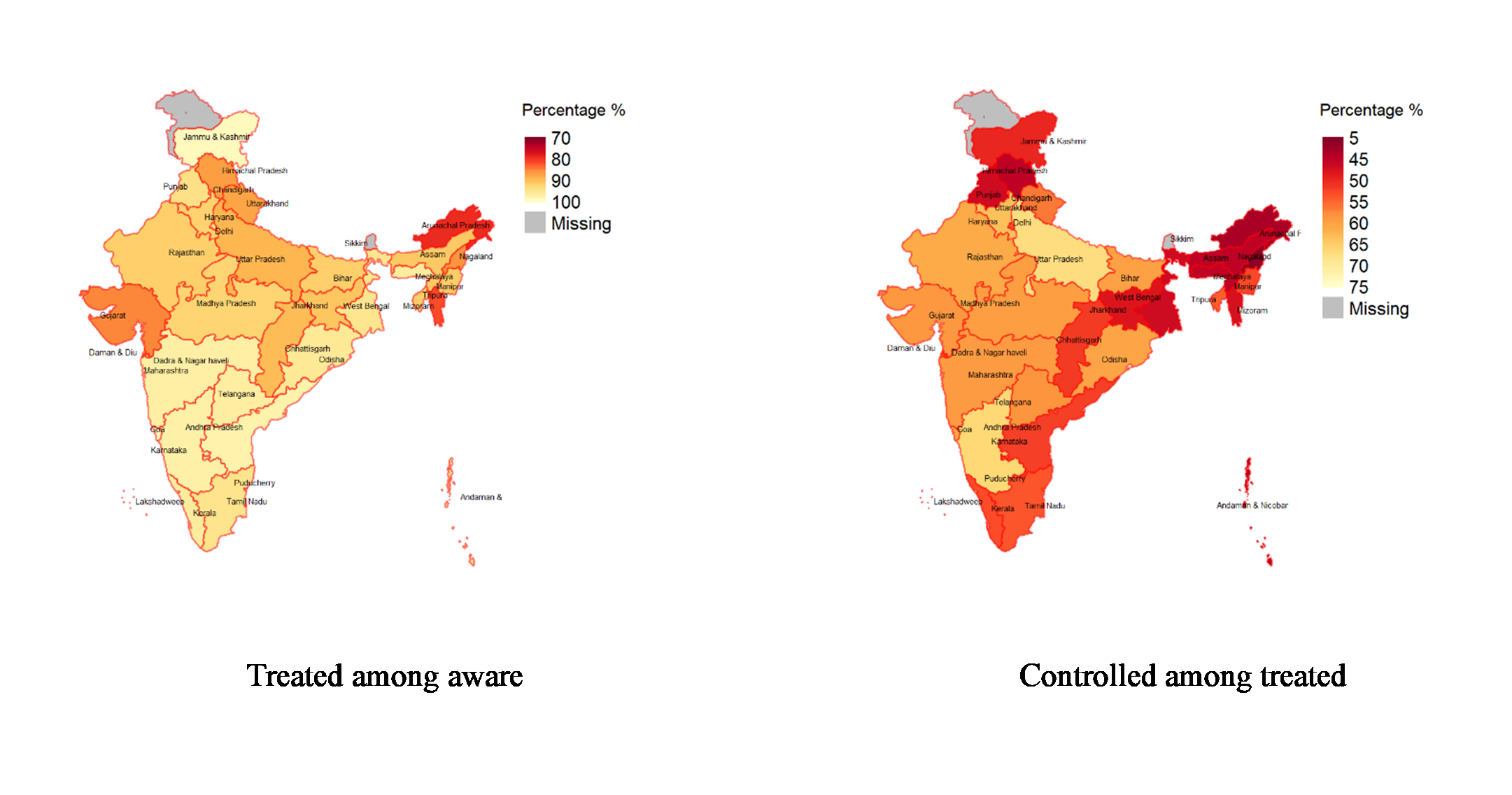

Supplement: S2 Fig — The base map can be found at https://globalsolaratlas.info/download/india. Adjusted for age and sex. (TIF) [file pmed.1003740.s007.tif]
