# Supplementary material for: Awareness, treatment, and control of hypertension in adults aged 45 years and over and their spouses in India: A nationally representative cross-sectional study
Source: PLoS Med. 2021 Aug 24;18(8):e1003740. doi: 10.1371/journal.pmed.1003740 (PMC8425529; doi:10.1371/journal.pmed.1003740)
Supplement: S1 Table — (DOCX) [file pmed.1003740.s008.docx]

**S1 Table. Sample characteristics by whether the blood pressure was measured, adults aged 45+ and their spouse, adults aged 45+ and their spouses**

|  | **Blood pressure measured** | | **P-value** |
| --- | --- | --- | --- |
|  | **Yes** | **No** |  |
|  | (n=65,751) | (n=6,499) |  |
|  | % | % |  |
| **MPCE quintile group** |  |  |  |
| Poorest | 20.3 | 17.5 | <0.001 |
| Poorer | 20.2 | 18.2 |  |
| Middle | 20.2 | 18.6 |  |
| Richer | 19.9 | 21.4 |  |
| Richest | 19.5 | 24.3 |  |
| **Education** |  |  |  |
| No schooling | 49.7 | 47.7 |  |
| < 5 years | 11.1 | 8.8 | <0.001 |
| 5-9 years | 21.4 | 19.2 |  |
| ≥ 10 years | 17.9 | 24.3 |  |
| **Age** |  |  |  |
| < 45 years | 8.8 | 7.7 | <0.001 |
| 45-54 | 31.7 | 33.9 |  |
| 55-64 | 27.7 | 23.5 |  |
| 65-74 | 22.0 | 19.5 |  |
| ≥75 | 9.9 | 15.5 |  |
| **Sex** |  |  |  |
| Male | 41.9 | 43.1 | <0.001 |
| Female | 58.1 | 56.9 |  |
| **Residence** |  |  |  |
| Rural | 69.5 | 56.8 | <0.001 |
| Urban | 30.5 | 43.2 |  |
| **Caste** |  |  |  |
| Scheduled caste | 19.4 | 16.9 | <0.001 |
| Scheduled tribe | 8.5 | 8.9 |  |
| Other Backward Class | 45.7 | 44.0 |  |
| Others | 26.3 | 30.2 |  |
| **Religion** |  |  |  |
| Hindu | 82.5 | 77.3 | 0.348 |
| Muslim | 11.2 | 15.6 |  |
| Christian | 2.9 | 3.7 |  |
| Others | 3.4 | 3.4 |  |
| **Marital status** |  |  |  |
| Currently married | 75.9 | 72.9 | <0.001 |
| Widowed | 21.4 | 23.6 |  |
| Others | 2.6 | 3.5 |  |
| **Living arrangement** |  |  |  |
| Living alone | 3.4 | 3.0 | <0.001 |
| Living with spouse and children | 15.3 | 21.2 |  |
| Living with children and others | 59.7 | 50.4 |  |
| Living with others only | 21.6 | 25.4 |  |
| **Working status** |  |  |  |
| Currently working | 46.9 | 41.3 | 0.004 |
| Worked but not currently | 25.5 | 31.5 |  |
| Never worked | 27.6 | 27.2 |  |
| **Health Insurance** |  |  |  |
| No | 79.1 | 81.2 | <0.001 |
| Yes | 20.9 | 18.8 |  |

MPCE- monthly per capita consumption expenditure
