# Supplementary material for: Awareness, treatment, and control of hypertension in adults aged 45 years and over and their spouses in India: A nationally representative cross-sectional study
Source: PLoS Med. 2021 Aug 24;18(8):e1003740. doi: 10.1371/journal.pmed.1003740 (PMC8425529; doi:10.1371/journal.pmed.1003740)
Supplement: S2 Table — (DOCX) [file pmed.1003740.s009.docx]

**S2 Table. Missing observations on BP measurement, diagnosis, treatment and socio-demographic variables, adults aged 45+ and their spouses**

|  | Missing | | | | |
| --- | --- | --- | --- | --- | --- |
|  | (n= 72,250) | |  | (n=65,751) | |
|  | **n** | **%** |  | n | % |
| BP measurement | 6499 | 9.00 |  | NA | NA |
| Reported hypertension/high BP diagnosis | 192 | 0.27 |  | 19 | 0.03 |
| Reported taking medication to control BP^a^ | 5 | 0.03 |  | 3 | 0.02 |
| Reported salt/diet restriction to control BP^b^ | 4 | 0.02 |  | 2 | 0.01 |
| MPCE quintile group | 1428 | 1.98 |  | 1088 | 1.65 |
| Education | 5 | 0.01 |  | 1 | 0.00 |
| Age | 0 | 0.00 |  | 0 | 0.00 |
| Sex | 0 | 0.00 |  | 0 | 0.00 |
| Location | 0 | 0.00 |  | 0 | 0.00 |
| Caste | 113 | 0.16 |  | 90 | 0.14 |
| Religion | 5 | 0.01 |  | 3 | 0.00 |
| Marital status | 4 | 0.01 |  | 2 | 0.00 |
| Living arrangement | 0 | 0.00 |  | 0 | 0.00 |
| Working status | 20 | 0.03 |  | 4 | 0.01 |
| Health Insurance | 729 | 1.01 |  | 139 | 0.21 |

^a, b^  Number of missing cases in based on those who reported ever diagnosed with high blood pressure (n was 19,877 and 18,048 for first and third column respectively)

NA: Not applicable
