# Supplementary material for: Awareness, treatment, and control of hypertension in adults aged 45 years and over and their spouses in India: A nationally representative cross-sectional study
Source: PLoS Med. 2021 Aug 24;18(8):e1003740. doi: 10.1371/journal.pmed.1003740 (PMC8425529; doi:10.1371/journal.pmed.1003740)
Supplement: S3 Table — (DOCX) [file pmed.1003740.s010.docx]

**S3 Table. Estimates of hypertension prevalence and rates of awareness, treatment, and control from full item response analysis sample and alternative sample including participants missing on MPCE or any sociodemographic variable, adults aged 45+ and their spouses**

|  | Full item response analysis sample  (n=64,427)  % (95% CI) | Sample including missings on covariates  (n=65,751)  % (95% CI) |
| --- | --- | --- |
| Hypertension prevalence | 41.9 (41.0-42.9) | 42.4 (41.2-43.6) |
|  |  |  |
| If hypertension | (n=28,600) | (n=29,263) |
| Awareness | 54.4 (53.1-55.7) | 54.6 (53.2-56.0) |
| Treatment | 50.8 (49.5-52.0) | 51.0 (49.6-52.4) |
| Control | 28.8 (27.4-30.1) | 28.7 (27.4-30.0) |
