# Supplementary material for: Awareness, treatment, and control of hypertension in adults aged 45 years and over and their spouses in India: A nationally representative cross-sectional study
Source: PLoS Med. 2021 Aug 24;18(8):e1003740. doi: 10.1371/journal.pmed.1003740 (PMC8425529; doi:10.1371/journal.pmed.1003740)
Supplement: S4 Table — (DOCX) [file pmed.1003740.s011.docx]

**S4 Table. Number of participants with hypertension by sociodemographic characteristics**

|  | **N** |
| --- | --- |
| **MPCE quintile group** |  |
| Poorest | 4,223 |
| Poorer | 4,750 |
| Middle | 5,384 |
| Richer | 6,545 |
| Richest | 7,698 |
| **Education attainment** |  |
| No schooling | 12,480 |
| < 5 years | 3,316 |
| 5-9 years | 6,863 |
| ≥ 10 years | 5,941 |
| **Age** |  |
| < 45 years | 1,439 |
| 45-54 | 7,924 |
| 55-64 | 8,655 |
| 65-74 | 7,218 |
| ≥75 | 3,364 |
| **Sex** |  |
| Male | 12,254 |
| Female | 16,346 |
| **Location** |  |
| Rural | 17,017 |
| Urban | 11,583 |
| **Caste** |  |
| Scheduled caste | 4,539 |
| Scheduled tribe | 4,847 |
| Other Backward Class | 10,446 |
| Others | 8,768 |
| **Religion** |  |
| Hindu | 20,216 |
| Muslim | 3,760 |
| Christian | 2,991 |
| Others | 1,633 |
| **Marital status** |  |
| Married | 20,585 |
| Widowed | 7,136 |
| Others | 879 |
| **Living arrangement** |  |
| Alone | 1,163 |
| With spouse | 4,412 |
| With children | 15,818 |
| With others | 7,207 |
| **Working status** |  |
| Working | 11,170 |
| Previously worked | 8,446 |
| Never worked | 8,984 |
| **Health Insurance** |  |
| No | 22,177 |
| Yes | 6,423 |
| **Total** | **28,600** |

MPCE- monthly per capita consumption expenditure
