# Supplementary material for: Awareness, treatment, and control of hypertension in adults aged 45 years and over and their spouses in India: A nationally representative cross-sectional study
Source: PLoS Med. 2021 Aug 24;18(8):e1003740. doi: 10.1371/journal.pmed.1003740 (PMC8425529; doi:10.1371/journal.pmed.1003740)
Supplement: S5 Table — (DOCX) [file pmed.1003740.s012.docx]

**S5 Table. Number of participants in full analysis sample and with hypertension by state**

| **State** | **Full analysis sample, N** | **With hypertension, N** |
| --- | --- | --- |
| Andaman & Nicobar Islands | 1,128 | 684 |
| Andhra Pradesh | 2,171 | 1,139 |
| Arunachal Pradesh | 1,142 | 426 |
| Assam | 2,114 | 908 |
| Bihar | 3,400 | 1195 |
| Chandigarh | 854 | 476 |
| Chhattisgarh | 1,880 | 814 |
| Dadra & Nagar Haveli | 875 | 344 |
| Daman & Diu | 837 | 390 |
| Delhi | 1,265 | 579 |
| Goa | 1224 | 642 |
| Gujarat | 1,980 | 812 |
| Haryana | 1,677 | 753 |
| Himachal Pradesh | 1,272 | 616 |
| Jammu & Kashmir | 1,389 | 661 |
| Jharkhand | 2,286 | 924 |
| Karnataka | 2,158 | 900 |
| Kerala | 2,176 | 1,252 |
| Lakshadweep | 1,013 | 663 |
| Madhya Pradesh | 2,604 | 902 |
| Maharashtra | 3,360 | 1,622 |
| Manipur | 1186 | 524 |
| Meghalaya | 881 | 441 |
| Mizoram | 1,110 | 380 |
| Nagaland | 1217 | 561 |
| Odisha | 2,641 | 958 |
| Puducherry | 1291 | 621 |
| Punjab | 1,914 | 1165 |
| Rajasthan | 2,066 | 781 |
| Tamil Nadu | 3,271 | 1502 |
| Telangana | 2,050 | 990 |
| Tripura | 1,073 | 458 |
| Uttar Pradesh | 4,142 | 1331 |
| Uttarakhand | 1,265 | 585 |
| West Bengal | 3,515 | 1,601 |
| **Total** | **64,427** | **28,600** |
