# Supplementary material for: Awareness, treatment, and control of hypertension in adults aged 45 years and over and their spouses in India: A nationally representative cross-sectional study
Source: PLoS Med. 2021 Aug 24;18(8):e1003740. doi: 10.1371/journal.pmed.1003740 (PMC8425529; doi:10.1371/journal.pmed.1003740)
Supplement: S6 Table — (DOCX) [file pmed.1003740.s013.docx]

**S6 Table. Unadjusted estimates of hypertension prevalence and percent aware, treated and controlled among those with hypertension by sociodemographic characteristics, adults aged 45+ and their spouses**

|  | **Prevalence**  **(N=64,427)** | **Awareness**  **(N=28,600)** | **Treatment**  **(N=28,600)** | **Control**  **(N=28,600)** |
| --- | --- | --- | --- | --- |
| **Overall** | **41.9 (41.0-42.9)** | **54.4 (53.1-55.7)** | **50.8 (49.5-52.0)** | **28.8 (27.4-30.1)** |
| **MPCE quintile** |  |  |  |  |
| Poorest | 36.1 (34.5-37.7) | 44.5 (41.9-47.1) | 40.9 (38.3-43.4) | 22.3 (20.1-24.5) |
| Poorer | 40.0 (38.4-41.5) | 50.1 (47.6-52.6) | 45.8 (43.3-48.4) | 24.6 (22.7-26.4) |
| Middle | 40.8 (39.3-42.4) | 54.8 (52.4-57.1) | 50.7 (48.4-53.1) | 28.0 (26.3-29.8) |
| Richer | 43.1 (41.6-44.7) | 58.9 (56.8-61.1) | 55.3 (53.2-57.5) | 32.2 (30.0-34.4) |
| Richest | 49.7 (47.2-52.2) | 60.7 (57.5-63.9) | 58.0 (55.0-60.9) | 34.4 (31.9-37.0) |
| **Education attainment** |  |  |  |  |
| No schooling | 39.7 (38.7-40.7) | 50.5 (48.7-52.2) | 46.6 (44.9-48.3) | 26.1 (24.7-27.5) |
| < 5 years | 42.6 (40.6-44.7) | 55.1 (52.1-58.2) | 51.1 (48.1-54.2) | 28.4 (25.9-31.0) |
| 5-9 years | 42.5 (41.0-44.0) | 56.9 (54.6-59.2) | 53.9 (51.6-56.2) | 29.9 (27.9-32.0) |
| ≥ 10 years | 47.3 (45.2-49.4) | 60.4 (56.7-64.2) | 57.0 (53.6-60.4) | 34.0 (31.3-36.7) |
| **Age** |  |  |  |  |
| < 45 years | 23.6 (19.6-27.6) | 45.1 (35.6-54.7) | 41.8 (32.9-50.8) | 27.4 (21.3-33.6) |
| 45-54 | 34.3 (33.1-35.6) | 47.8 (45.1-50.5) | 44.3 (41.7-46.8) | 26.5 (24.4-28.6) |
| 55-64 | 44.1 (42.7-45.4) | 55.2 (52.8-57.5) | 52.0 (49.5-54.4) | 29.7 (27.5-31.9) |
| 65-74 | 52.2 (50.4-54.0) | 59.2 (56.7-61.7) | 55.6 (53.0-58.2) | 30.1 (27.0-33.2) |
| ≥75 | 54.2 (52.1-56.3) | 59.1 (56.2-62.1) | 54.4 (51.4-57.5) | 28.7 (25.6-31.8) |
| **Sex** |  |  |  |  |
| Male | 41.7 (40.5-42.9) | 48.8 (47.1-50.4) | 45.0 (43.4-46.7) | 24.5 (23.2-25.9) |
| Female | 42.1 (41.1-43.1) | 58.4 (56.9-59.9) | 54.9 (53.4-56.4) | 31.8 (30.2-33.4) |
| **Location** |  |  |  |  |
| Rural | 38.1 (37.2-38.9) | 49.6 (48.0-51.2) | 45.8 (44.3-47.4) | 25.4 (24.1-26.7) |
| Urban | 51.2 (49.7-52.6) | 62.7 (60.4-65.0) | 59.5 (57.4-61.7) | 34.6 (32.4-36.9) |
| **Caste** |  |  |  |  |
| Scheduled caste | 38.7 (37.3-40.0) | 52.0 (49.5-54.4) | 47.9 (45.4-50.4) | 26.5 (24.2-28.7) |
| Scheduled tribe | 36.5 (34.0-39.0) | 35.4 (31.9-38.9) | 31.7 (28.5-35.0) | 16.7 (14.1-19.2) |
| Other Backward Class | 41.9 (40.4-43.5) | 54.1 (52.2-56.0) | 50.8 (48.9-52.6) | 29.5 (27.3-31.8) |
| Others | 46.2 (44.8-47.6) | 61.2 (59.3-63.0) | 57.5 (55.6-59.3) | 32.1 (30.4-33.7) |
| **Religion** |  |  |  |  |
| Hindu | 40.9 (39.7-42.1) | 53.1 (51.6-54.6) | 49.5 (48.0-50.9) | 28.4 (26.8-29.9) |
| Muslim | 46.1 (43.3-49.0) | 60.4 (57.6-63.2) | 56.8 (54.1-59.6) | 31.8 (29.2-34.5) |
| Christian | 43.2 (36.5-49.9) | 54.6 (49.7-59.5) | 51.5 (46.6-56.4) | 27.2 (23.0-31.3) |
| Others | 52.0 (48.2-55.7) | 60.6 (56.1-65.1) | 56.9 (52.6-61.2) | 27.8 (24.6-31.1) |
| **Marital status** |  |  |  |  |
| Married | 38.9 (37.9-40.0) | 53.0 (51.1-54.8) | 49.5 (47.7-51.3) | 28.6 (27.3-29.8) |
| Widowed | 53.3 (51.6-55.1) | 58.8 (56.4-61.3) | 54.8 (52.3-57.3) | 29.8 (26.8-32.9) |
| Others | 37.2 (30.4-44.1) | 45.5 (39.2-51.8) | 43.0 (37.1-48.9) | 21.7 (17.1-26.3) |
| **Living arrangement** |  |  |  |  |
| Alone | 52.5 (48.9-56.0) | 55.1 (50.8-59.5) | 51.5 (47.2-55.9) | 27.5 (23.6-31.4) |
| With spouse | 43.1 (40.7-45.6) | 54.3 (51.6-57.1) | 50.9 (48.1-53.7) | 28.2 (26.0-30.4) |
| With children | 37.8 (36.4-39.2) | 52.5 (50.4-54.5) | 49.0 (47.0-50.9) | 28.6 (27.2-30.0) |
| With others | 51.0 (49.6-52.4) | 58.2 (55.7-60.7) | 54.3 (51.7-56.8) | 29.6 (26.4-32.7) |
| **Working status** |  |  |  |  |
| Working | 35.9 (34.3-37.4) | 42.4 (40.2-44.5) | 39.1 (37.1-41.1) | 22.6 (21.2-23.9) |
| Previously worked | 50.3 (48.8-51.8) | 60.3 (58.6-62.1) | 56.2 (54.5-57.9) | 30.3 (28.5-32.1) |
| Never worked | 44.6 (43.2-45.9) | 64.7 (62.3-67.1) | 61.3 (58.7-63.8) | 35.7 (32.4-38.9) |
| **Health Insurance** |  |  |  |  |
| No | 41.8 (40.9-42.7) | 54.5 (53.1-56.0) | 50.8 (49.4-52.3) | 28.9 (27.4-30.4) |
| Yes | 42.5 (40.6-44.3) | 53.8 (51.5-56.2) | 50.5 (48.3-52.8) | 28.3 (26.4-30.1) |
