# Supplementary material for: Awareness, treatment, and control of hypertension in adults aged 45 years and over and their spouses in India: A nationally representative cross-sectional study
Source: PLoS Med. 2021 Aug 24;18(8):e1003740. doi: 10.1371/journal.pmed.1003740 (PMC8425529; doi:10.1371/journal.pmed.1003740)
Supplement: S7 Table — (DOCX) [file pmed.1003740.s014.docx]

**S7 Table. Adjusted prevalence of undiagnosed, untreated and uncontrolled hypertension by MPCE quintile and education groups, adults aged 45+ and their spouses in India**

|  | **n=64,427** | | | | |  |
| --- | --- | --- | --- | --- | --- | --- |
|  | **Undiagnosed (95% CI)** | **F-statistic (p-value)** | **Untreated (95% CI)** | **F-statistic (p-value)** | **Uncontrolled (95% CI)** | **F-statistic (p-value)** |
| **Overall** | 19.1 (18.5-19.8) |  | 20.7 (20.0-21.3) |  | 29.9 (29.2-30.6) |  |
| **MPCE quintile group** |  |  |  |  |  |  |
| Poorest | 19.9 (18.5-21.2) | 2.36 (0.0524) | 21.1 (19.8-22.5) | 2.09 (0.0802) | 27.5 (25.9-29.2) | 5.95 (0.0001) |
| Poorer | 19.8 (18.7-21.0) |  | 21.5 (20.3-22.7) |  | 29.8 (28.5-31.2) |  |
| Middle | 18.5 (17.4-19.5) |  | 20.1 (19.1-21.2) |  | 29.5 (28.2-30.7) |  |
| Richer | 17.7 (16.7-18.8) |  | 19.3 (18.2-20.4) |  | 29.4 (28.0-30.8) |  |
| Richest | 19.8 (17.8-21.7) |  | 21.2 (19.4-22.9) |  | 33.3 (31.7-34.9) |  |
| **Education** |  |  |  |  |  |  |
| No schooling | 19.8 (18.9-20.7) | 1.67 (0.1717) | 21.2 (20.4-22.1) | 1.51 (0.2089) | 28.2 (27.3-29.1) | 9.48 (0.0000) |
| 1-4 years | 18.9 (17.4-20.3) |  | 20.6 (19.1-22.0) |  | 30.4 (28.7-32.2) |  |
| 5-9 years | 18.4 (17.3-19.5) |  | 19.8 (18.6-20.9) |  | 31.3 (29.9-32.8) |  |
| >=10 years | 18.4 (16.2-20.5) |  | 20.1 (18.1-22.2) |  | 32.8 (31.4-34.3) |  |

“Undiagnosed” and “Untreated” show percent of full analysis sample with measured high blood pressure (≥140/90 mm Hg) and with no reported diagnosis or treatment, respectively, of hypertension. “Uncontrolled” shows percent of full analysis sample with measured high blood pressure (≥140/90 mm Hg). Prevalence rates by MPCE quintile group and education groups are adjusted for age and sex. F-statistic for test of equal prevalence across groups defined by each characteristic. MPCE- monthly per capita consumption expenditure

|  | **(N=64,427)** | **(N=28,600)** |
| --- | --- | --- |
