# Supplementary material for: Awareness, treatment, and control of hypertension in adults aged 45 years and over and their spouses in India: A nationally representative cross-sectional study
Source: PLoS Med. 2021 Aug 24;18(8):e1003740. doi: 10.1371/journal.pmed.1003740 (PMC8425529; doi:10.1371/journal.pmed.1003740)
Supplement: S8 Table — (DOCX) [file pmed.1003740.s015.docx]

**S8 Table. Adjusted hypertension prevalence, and percent aware, treated, and controlled among those with hypertension by state, adults aged 45+ and their spouses in India**

|  | **N=64,427** |  | **(N=28,600)** |  |
| --- | --- | --- | --- | --- |
|  | **Prevalence** | **Awareness** | **Treatment** | **Control** |
|  | **% (95% CI)** | **% (95% CI)** | **% (95% CI)** | **% (95% CI)** |
| **India** | **41.9 (41.0-42.9)** | **54.4 (53.1-55.7)** | **50.8 (49.5-52.0)** | **28.8 (27.4-30.1)** |
| **State** |  |  |  |  |
| Andaman & Nicobar Islands | 60.8 (55.2-66.4) | 64.9 (58.6-71.1) | 60.7 (55.1-66.2) | 25.5 (20.3-30.6) |
| Andhra Pradesh | 54.3 (51.7-57.0) | 60.8 (57.4-64.3) | 59.1 (55.9-62.4) | 30.5 (27.3-33.7) |
| Arunachal Pradesh | 43.3 (38.3-48.3) | 42.9 (34.6-51.1) | 32.9 (24.0-41.8) | 10.7 (6.7-14.8) |
| Assam | 46.4 (43.5-49.3) | 61.0 (56.7-65.3) | 55.5 (51.3-59.7) | 24.8 (20.3-29.2) |
| Bihar | 35.3 (33.0-37.6) | 52.4 (46.8-58.0) | 47.9 (43.0-52.7) | 28.6 (23.3-34.0) |
| Chandigarh | 57.4 (52.7-62.1) | 69.6 (62.3-76.9) | 68.1 (60.9-75.3) | 41.0 (32.8-49.1) |
| Chhattisgarh | 44.0 (40.9-47.2) | 32.3 (27.1-37.5) | 28.9 (23.6-34.3) | 14.7 (11.2-18.3) |
| Dadra & Nagar Haveli | 43.7 (39.4-48.0) | 40.1 (30.1-50.0) | 36.8 (26.7-46.9) | 19.1 (11.8-26.3) |
| Daman & Diu | 48.0 (44.2-51.8) | 56.9 (49.0-64.8) | 52.1 (43.9-60.4) | 27.8 (20.2-35.4) |
| Delhi | 50.8 (47.2-54.4) | 67.4 (62.0-72.8) | 63.3 (58.0-68.7) | 34.0 (28.6-39.5) |
| Goa | 54.9 (51.5-58.2) | 72.3 (67.8-76.8) | 71.2 (66.6-75.8) | 41.9 (36.8-46.9) |
| Gujarat | 42.5 (38.9-46.0) | 47.4 (41.5-53.2) | 40.2 (34.9-45.4) | 23.3 (20.0-26.7) |
| Haryana | 45.7 (41.9-49.5) | 68.6 (64.6-72.5) | 62.7 (58.6-66.7) | 39.0 (34.1-43.8) |
| Himachal Pradesh | 50.2 (45.7-54.6) | 55.5 (50.1-61.0) | 48.5 (43.9-53.2) | 20.4 (16.4-24.4) |
| Jammu & Kashmir | 48.7 (44.3-53.1) | 75.9 (70.8-81.1) | 74.9 (69.8-79.9) | 36.9 (29.8-43.9) |
| Jharkhand | 41.2 (38.4-44.0) | 45.3 (40.9-49.8) | 41.1 (36.7-45.5) | 19.7 (16.6-22.8) |
| Karnataka | 42.9 (37.9-47.9) | 49.1 (44.2-54.1) | 46.8 (41.4-52.3) | 28.5 (21.2-35.8) |
| Kerala | 57.0 (54.0-60.0) | 65.2 (62.2-68.1) | 61.7 (58.3-65.1) | 30.7 (27.7-33.6) |
| Lakshadweep | 66.0 (61.3-70.6) | 54.8 (49.1-60.5) | 51.3 (46.2-56.4) | 16.1 (11.4-20.8) |
| Madhya Pradesh | 35.0 (31.9-38.1) | 44.1 (35.1-53.0) | 40.6 (32.3-48.8) | 24.1 (18.1-30.1) |
| Maharashtra | 48.5 (45.8-51.3) | 52.9 (49.1-56.7) | 51.1 (47.1-55.0) | 30.0 (26.1-33.8) |
| Manipur | 43.2 (38.0-48.3) | 54.4 (45.4-63.4) | 48.8 (39.9-57.7) | 24.5 (19.3-29.7) |
| Meghalaya | 50.9 (44.7-57.1) | 51.0 (41.4-60.7) | 48.9 (39.5-58.4) | 22.1 (16.6-27.7) |
| Mizoram | 33.1 (29.4-36.8) | 64.2 (57.8-70.6) | 54.0 (48.5-59.5) | 26.4 (21.5-31.4) |
| Nagaland | 53.4 (43.9-62.9) | 27.5 (22.2-32.8) | 23.8 (17.6-30.1) | 4.6 (1.1-8.1) |
| Odisha | 36.4 (33.6-39.1) | 47.5 (42.5-52.4) | 45.1 (40.4-49.9) | 26.9 (23.2-30.6) |
| Puducherry | 47.4 (44.4-50.5) | 64.3 (59.8-68.9) | 63.3 (58.8-67.7) | 41.1 (35.0-47.2) |
| Punjab | 60.2 (57.7-62.7) | 67.6 (63.7-71.4) | 63.6 (60.2-67.0) | 29.5 (26.6-32.4) |
| Rajasthan | 36.8 (33.9-39.7) | 58.1 (53.3-63.0) | 53.1 (48.7-57.5) | 32.6 (29.4-35.7) |
| Tamil Nadu | 42.9 (40.6-45.2) | 55.4 (51.7-59.0) | 51.9 (48.2-55.5) | 28.2 (24.9-31.5) |
| Telangana | 48.8 (46.0-51.5) | 62.1 (58.3-65.8) | 59.7 (55.9-63.5) | 34.8 (31.6-38.1) |
| Tripura | 45.1 (41.6-48.6) | 58.5 (50.7-66.3) | 53.4 (45.9-61.0) | 28.8 (23.0-34.6) |
| Uttar Pradesh | 31.3 (29.2-33.5) | 51.0 (47.4-54.5) | 45.4 (41.8-49.0) | 30.7 (27.8-33.5) |
| Uttarakhand | 44.9 (40.4-49.5) | 51.1 (44.2-58.0) | 44.7 (37.7-51.6) | 25.2 (18.7-31.8) |
| West Bengal | 44.4 (41.2-47.6) | 63.4 (59.8-66.9) | 60.2 (56.4-64.0) | 28.3 (25.1-31.6) |

Adjusted for age and sex. See S5 Table for state-specific sample sizes
