# Supplementary material for: Awareness, treatment, and control of hypertension in adults aged 45 years and over and their spouses in India: A nationally representative cross-sectional study
Source: PLoS Med. 2021 Aug 24;18(8):e1003740. doi: 10.1371/journal.pmed.1003740 (PMC8425529; doi:10.1371/journal.pmed.1003740)
Supplement: S9 Table — (DOCX) [file pmed.1003740.s016.docx]

**S9 Table. Unadjusted estimates of hypertension prevalence and percent aware, treated, and controlled among those with hypertension by state, adults aged 45+ and their spouses in India**

|  | **Prevalence** | **Awareness** | **Treatment** | **Control** |
| --- | --- | --- | --- | --- |
|  | **% (95% CI)** | **% (95% CI)** | **% (95% CI)** | **% (95% CI)** |
| **India** | **41.9 (41.0-42.9)** | **54.4 (53.1-55.7)** | **50.8 (49.5-52.0)** | **28.8 (27.4-30.1)** |
| **States/UTs** |  |  |  |  |
| Andaman & Nicobar Islands | 59.2 (53.3-65.1) | 63.4 (56.8-70.0) | 59.1 (53.1-65.1) | 24.9 (19.9-29.9) |
| Andhra Pradesh | 53.1 (50.5-55.8) | 60.2 (56.6-63.7) | 58.5 (55.0-61.9) | 30.3 (27.1-33.4) |
| Arunachal Pradesh | 37.2 (32.2-42.2) | 38.1 (30.4-45.9) | 28.8 (20.8-36.9) | 9.8 (6.0-13.7) |
| Assam | 43.4 (40.5-46.3) | 60.1 (55.6-64.5) | 54.5 (50.2-58.9) | 24.7 (20.2-29.2) |
| Bihar | 36.6 (34.0-39.1) | 53.1 (47.5-58.7) | 48.6 (43.7-53.5) | 28.7 (23.0-34.4) |
| Chandigarh | 56.4 (51.2-61.6) | 69.2 (61.5-76.9) | 67.7 (60.1-75.3) | 41.0 (33.0-49.0) |
| Chhattisgarh | 42.7 (39.4-45.9) | 31.1 (25.9-36.4) | 27.9 (22.6-33.3) | 14.6 (11.1-18.2) |
| Dadra & Nagar Haveli | 41.9 (37.4-46.5) | 37.5 (28.1-47.0) | 34.4 (24.8-44.0) | 18.0 (11.2-24.9) |
| Daman & Diu | 48.1 (44.5-51.7) | 57.5 (50.0-65.0) | 52.7 (44.9-60.5) | 28.1 (20.8-35.5) |
| Delhi | 47.9 (44.1-51.7) | 65.2 (59.1-71.2) | 61.0 (55.0-66.9) | 32.9 (27.4-38.3) |
| Goa | 54.8 (51.2-58.3) | 72.8 (68.4-77.2) | 71.8 (67.3-76.3) | 42.0 (37.0-47.0) |
| Gujarat | 42.6 (38.8-46.4) | 47.6 (41.7-53.6) | 40.5 (35.2-45.8) | 23.4 (20.0-26.8) |
| Haryana | 46.4 (42.6-50.3) | 68.3 (64.2-72.3) | 62.3 (58.3-66.3) | 38.8 (34.1-43.5) |
| Himachal Pradesh | 50.1 (45.7-54.6) | 55.7 (50.2-61.1) | 48.6 (44.0-53.3) | 20.6 (16.4-24.8) |
| Jammu & Kashmir | 49.4 (45.2-53.5) | 76.9 (71.9-81.9) | 75.8 (70.9-80.7) | 37.7 (30.5-44.9) |
| Jharkhand | 41.1 (38.2-44.0) | 45.3 (40.9-49.7) | 41.1 (36.7-45.5) | 19.6 (16.6-22.7) |
| Karnataka | 41.6 (36.9-46.2) | 48.8 (43.7-53.8) | 46.4 (41.1-51.8) | 28.4 (21.4-35.5) |
| Kerala | 58.5 (55.4-61.7) | 66.4 (63.5-69.3) | 62.9 (59.5-66.3) | 31.0 (27.9-34.0) |
| Lakshadweep | 66.9 (61.7-72.1) | 55.5 (50.3-60.7) | 52.1 (47.2-56.9) | 16.5 (11.7-21.3) |
| Madhya Pradesh | 34.9 (31.9-38.0) | 43.9 (34.4-53.4) | 40.4 (31.5-49.3) | 24.0 (17.8-30.2) |
| Maharashtra | 49.1 (46.3-51.9) | 53.1 (49.3-56.8) | 51.3 (47.3-55.2) | 30.1 (26.2-33.9) |
| Manipur | 43.8 (38.3-49.2) | 53.4 (44.3-62.5) | 47.8 (38.9-56.8) | 23.9 (18.9-29.0) |
| Meghalaya | 50.4 (43.7-57.1) | 51.1 (41.2-60.9) | 48.9 (39.2-58.6) | 22.2 (16.6-27.8) |
| Mizoram | 33.3 (29.7-37.0) | 63.4 (57.0-69.7) | 53.0 (47.0-59.0) | 25.5 (20.6-30.3) |
| Nagaland | 53.7 (44.5-62.8) | 27.8 (22.5-33.2) | 24.1 (17.8-30.4) | 4.6 (1.1-8.1) |
| Odisha | 35.9 (33.1-38.6) | 47.5 (42.4-52.5) | 45.2 (40.3-50.1) | 27.0 (23.2-30.7) |
| Puducherry | 48.0 (44.5-51.5) | 64.6 (59.9-69.2) | 63.5 (58.9-68.2) | 41.0 (35.0-47.0) |
| Punjab | 60.5 (57.9-63.1) | 67.2 (63.2-71.2) | 63.1 (59.6-66.7) | 29.3 (26.4-32.2) |
| Rajasthan | 38.1 (35.1-41.1) | 58.9 (54.0-63.8) | 53.8 (49.4-58.2) | 32.7 (29.5-35.9) |
| Tamil Nadu | 42.9 (40.6-45.2) | 55.5 (51.8-59.2) | 52.1 (48.4-55.8) | 28.3 (25.0-31.6) |
| Telangana | 48.7 (46.0-51.3) | 62.1 (58.1-66.1) | 59.8 (55.8-63.8) | 34.8 (31.4-38.1) |
| Tripura | 43.4 (39.7-47.0) | 57.6 (49.6-65.6) | 52.5 (44.7-60.3) | 28.4 (22.5-34.4) |
| Uttar Pradesh | 31.8 (29.7-34.0) | 51.1 (47.5-54.7) | 45.5 (41.9-49.1) | 30.7 (27.8-33.5) |
| Uttarakhand | 45.5 (40.8-50.2) | 50.7 (43.8-57.7) | 44.4 (37.4-51.3) | 25.3 (18.7-32.0) |
| West Bengal | 42.7 (39.5-45.8) | 63.0 (59.4-66.7) | 59.9 (56.0-63.8) | 28.3 (25.1-31.5) |
