# Supplementary material for: Awareness, treatment, and control of hypertension in adults aged 45 years and over and their spouses in India: A nationally representative cross-sectional study
Source: PLoS Med. 2021 Aug 24;18(8):e1003740. doi: 10.1371/journal.pmed.1003740 (PMC8425529; doi:10.1371/journal.pmed.1003740)
Supplement: S10 Table — (DOCX) [file pmed.1003740.s017.docx]

**S10 Table. Correlation of rates of ATC each with hypertension prevalence across states, adults aged 45+ and their spouses.**

|  | Spearman rank correlation  (p-value) | Pearson correlation  (p-value) |
| --- | --- | --- |
| Awareness | 0.512 (0.001) | 0.341 (0.044) |
| Treatment | 0.543 (<0.001) | 0.395 (0.018) |
| Control | 0.189 (0.277) | 0.040 (0.819) |

ATC: Awareness, treatment, and control
