# Supplementary material for: Awareness, treatment, and control of hypertension in adults aged 45 years and over and their spouses in India: A nationally representative cross-sectional study
Source: PLoS Med. 2021 Aug 24;18(8):e1003740. doi: 10.1371/journal.pmed.1003740 (PMC8425529; doi:10.1371/journal.pmed.1003740)
Supplement: S11 Table — (DOCX) [file pmed.1003740.s018.docx]

| **S11 Table. Adjusted concentration indices for hypertension and ATC among those with hypertension, adults aged 45+ and their spouses in India** | | | | |
| --- | --- | --- | --- | --- |
|  | **Prevalence** | **Awareness** | **Treatment** | **Control** |
|  | **Index (95 % CI)** | **Index (95 % CI)** | **Index (95 % CI)** | **Index (95 % CI)** |
| **India** | **0.061 (0.048-0.073)** | **0.072 (0.056-0.089)** | **0.077 (0.061-0.093)** | **0.055 (0.043-0.067)** |
| **States/UTs** |  |  |  |  |
| Andaman & Nicobar Islands | -0.029 (-0.074-0.016) | 0.049 (-0.020-0.119) | 0.060 (-0.015-0.136) | 0.032 (-0.019-0.082) |
| Andhra Pradesh | 0.010 (-0.016-0.037) | 0.039 (0.005-0.072) | 0.035 (0.001-0.069) | 0.018 (-0.014-0.049) |
| Arunachal Pradesh | 0.058 (0.017-0.098) | 0.102 (-0.004-0.209) | 0.098 (-0.005-0.201) | 0.026 (-0.024-0.075) |
| Assam | 0.024 (-0.002-0.051) | 0.095 (0.051-0.139) | 0.107 (0.064-0.150) | 0.041 (0.003-0.079) |
| Bihar | 0.010 (-0.034-0.055) | 0.094 (0.060-0.128) | 0.098 (0.063-0.133) | 0.090 (0.052-0.128) |
| Chandigarh | 0.029 (-0.022-0.080) | 0.050 (-0.022-0.122) | 0.057 (-0.011-0.125) | 0.093 (0.036-0.151) |
| Chhattisgarh | 0.054 (0.030-0.079) | 0.116 (0.072-0.159) | 0.122 (0.078-0.165) | 0.057 (0.019-0.095) |
| Dadra & Nagar Haveli | 0.084 (0.029-0.140) | 0.193 (0.123-0.264) | 0.189 (0.108-0.270) | 0.112 (0.073-0.151) |
| Daman & Diu | 0.017 (-0.041-0.076) | -0.023 (-0.110-0.064) | -0.037 (-0.126-0.052) | 0.025 (-0.044-0.094) |
| Delhi | 0.051 (0.017-0.085) | -0.001 (-0.101-0.098) | 0.014 (-0.084-0.112) | 0.001 (-0.110-0.112) |
| Goa | 0.039 (0.008-0.070) | 0.034 (-0.014-0.081) | 0.039 (-0.009-0.087) | -0.019 (-0.066-0.028) |
| Gujarat | 0.017 (-0.016-0.050) | 0.051 (0.002-0.100) | 0.065 (0.022-0.108) | 0.041 (0.001-0.080) |
| Haryana | 0.040 (0.001-0.079) | -0.024 (-0.066-0.018) | -0.038 (-0.081-0.004) | -0.046 (-0.089--0.003) |
| Himachal Pradesh | 0.030 (-0.010-0.070) | 0.051 (-0.009-0.112) | 0.046 (-0.013-0.105) | 0.015 (-0.020-0.050) |
| Jammu & Kashmir | 0.035 (-0.007-0.077) | 0.034 (-0.009-0.078) | 0.045 (-0.002-0.092) | 0.034 (-0.007-0.076) |
| Jharkhand | 0.057 (0.027-0.086) | 0.117 (0.071-0.163) | 0.126 (0.084-0.168) | 0.069 (0.040-0.097) |
| Karnataka | 0.119 (0.063-0.175) | 0.067 (-0.017-0.152) | 0.080 (-0.002-0.161) | 0.101 (0.069-0.133) |
| Kerala | 0.000 (-0.035-0.035) | -0.010 (-0.049-0.029) | -0.008 (-0.046-0.031) | 0.018 (-0.022-0.058) |
| Lakshadweep | -0.015 (-0.050-0.020) | 0.020 (-0.036-0.077) | 0.022 (-0.036-0.080) | 0.023 (-0.023-0.070) |
| Madhya Pradesh | -0.005 (-0.050-0.039) | 0.092 (0.014-0.170) | 0.093 (0.015-0.172) | 0.084 (0.046-0.121) |
| Maharashtra | 0.030 (0.002-0.059) | 0.049 (0.013-0.086) | 0.050 (0.012-0.089) | 0.053 (0.016-0.090) |
| Manipur | 0.002 (-0.053-0.058) | 0.024 (-0.033-0.082) | 0.054 (0.003-0.105) | 0.058 (0.021-0.095) |
| Meghalaya | 0.050 (-0.024-0.125) | 0.055 (-0.031-0.141) | 0.072 (-0.009-0.153) | 0.003 (-0.036-0.042) |
| Mizoram | 0.053 (0.013-0.093) | 0.059 (-0.023-0.142) | 0.074 (0.005-0.143) | 0.030 (-0.049-0.108) |
| Nagaland | 0.072 (0.031-0.114) | 0.117 (0.047-0.186) | 0.116 (0.047-0.185) | 0.036 (0.007-0.064) |
| Odisha | 0.068 (0.044-0.092) | 0.136 (0.091-0.181) | 0.131 (0.087-0.174) | 0.076 (0.034-0.118) |
| Puducherry | 0.021 (-0.017-0.059) | 0.028 (-0.027-0.082) | 0.029 (-0.027-0.086) | 0.022 (-0.064-0.108) |
| Punjab | 0.010 (-0.024-0.045) | 0.002 (-0.040-0.044) | 0.004 (-0.032-0.040) | 0.004 (-0.035-0.044) |
| Rajasthan | 0.044 (0.011-0.076) | 0.070 (0.026-0.114) | 0.059 (0.015-0.104) | 0.030 (-0.021-0.081) |
| Tamil Nadu | 0.034 (0.007-0.060) | 0.033 (-0.006-0.072) | 0.037 (-0.002-0.076) | 0.024 (-0.013-0.061) |
| Telangana | 0.041 (0.011-0.072) | 0.049 (0.011-0.086) | 0.059 (0.021-0.096) | 0.056 (0.010-0.102) |
| Tripura | 0.017 (-0.019-0.052) | 0.151 (0.096-0.206) | 0.148 (0.090-0.206) | 0.102 (0.057-0.148) |
| Uttar Pradesh | 0.052 (0.032-0.072) | 0.088 (0.054-0.122) | 0.088 (0.050-0.126) | 0.046 (0.008-0.084) |
| Uttarakhand | 0.031 (-0.015-0.077) | 0.080 (0.016-0.143) | 0.093 (0.036-0.151) | 0.077 (0.021-0.133) |
| West Bengal | 0.075 (0.049-0.101) | 0.043 (0.004-0.082) | 0.052 (0.011-0.093) | 0.044 (0.009-0.080) |

ATC: Awareness, treatment, and control

A concentration index is a scaled covariance between an outcome and rank in the distribution of MPCE. Adjusted for age and sex. See S5 Table for state-specific sample sizes. MPCE, monthly per capita consumption expenditure
