# Supplementary material for: Awareness, treatment, and control of hypertension in adults aged 45 years and over and their spouses in India: A nationally representative cross-sectional study
Source: PLoS Med. 2021 Aug 24;18(8):e1003740. doi: 10.1371/journal.pmed.1003740 (PMC8425529; doi:10.1371/journal.pmed.1003740)
Supplement: S12 Table — (DOCX) [file pmed.1003740.s019.docx]

**S12 Table. Adjusted marginal effect for hypertension, and ATC among those with hypertension, adults aged 45+ and their spouses in India. ATC: Awareness, treatment, and control**

|  | **(N=64,427)** | **(N=28,600)** | | |
| --- | --- | --- | --- | --- |
|  | **Prevalence** | **Awareness** | **Treatment** | **Control** |
|  | **Marginal effect (95% CI)** | **Marginal effect (95% CI)** | **Marginal effect (95% CI)** | **Marginal effect (95% CI)** |
| **MPCE quintile group** |  |  |  |  |
| Poorest | ref. | ref. | ref. | ref. |
| Poorer | 0.023 (0.003-0.042) | 0.038 (0.008-0.068) | 0.032 (0.001-0.062) | 0.014 (-0.013-0.040) |
| Middle | 0.023 (0.000-0.047) | 0.069 (0.040-0.097) | 0.064 (0.036-0.092) | 0.041 (0.016-0.066) |
| Richer | 0.025 (0.002-0.048) | 0.089 (0.056-0.121) | 0.087 (0.055-0.119) | 0.070 (0.040-0.099) |
| Richest | 0.061 (0.030-0.092) | 0.085 (0.053-0.117) | 0.089 (0.057-0.120) | 0.071 (0.042-0.101) |
| **Education** |  |  |  |  |
| No schooling | ref. | ref. | ref. | ref. |
| < 5 years | 0.032 (0.010-0.053) | 0.077 (0.044-0.110) | 0.072 (0.039-0.106) | 0.045 (0.016-0.073) |
| 5-9 years | 0.049 (0.032-0.066) | 0.082 (0.052-0.112) | 0.090 (0.060-0.119) | 0.044 (0.019-0.070) |
| ≥ 10 years | 0.069 (0.045-0.093) | 0.118 (0.088-0.148) | 0.118 (0.088-0.148) | 0.074 (0.042-0.106) |
| **Age** |  |  |  |  |
| < 45 years | ref. | ref. | ref. | ref. |
| 45-54 | 0.131 (0.095-0.166) | 0.109 (0.032-0.186) | 0.108 (0.035-0.181) | 0.045 (-0.016-0.106) |
| 55-64 | 0.228 (0.191-0.265) | 0.173 (0.074-0.271) | 0.177 (0.082-0.272) | 0.074 (-0.012-0.160) |
| 65-74 | 0.290 (0.262-0.319) | 0.193 (0.094-0.291) | 0.196 (0.101-0.290) | 0.071 (-0.020-0.163) |
| ≥75 | 0.299 (0.262-0.337) | 0.184 (0.087-0.280) | 0.177 (0.084-0.270) | 0.059 (-0.030-0.148) |
| **Sex** |  |  |  |  |
| Male | ref. | ref. | ref. | ref. |
| Female | 0.028 (0.012-0.043) | 0.108 (0.083-0.134) | 0.111 (0.086-0.137) | 0.077 (0.055-0.098) |
| **Residence** |  |  |  |  |
| Rural | ref. | ref. | ref. | ref. |
| Urban | 0.084 (0.066-0.101) | 0.082 (0.056-0.107) | 0.081 (0.058-0.105) | 0.046 (0.025-0.067) |
| **Caste** |  |  |  |  |
| Scheduled caste | ref. | ref. | ref. | ref. |
| Scheduled tribe | -0.013 (-0.045-0.019) | -0.106 (-0.150--0.061) | -0.103 (-0.146--0.061) | -0.069 (-0.108--0.030) |
| Other Backward Class | 0.000 (-0.016-0.017) | 0.004 (-0.026-0.034) | 0.006 (-0.025-0.036) | 0.002 (-0.025-0.030) |
| Others | 0.010 (-0.008-0.028) | 0.006 (-0.026-0.038) | 0.006 (-0.027-0.040) | 0.000 (-0.030-0.030) |
| **Religion** |  |  |  |  |
| Hindu | ref. | ref. | ref. | ref. |
| Muslim | 0.052 (0.025-0.080) | 0.045 (0.015-0.075) | 0.044 (0.015-0.073) | 0.022 (-0.005-0.050) |
| Christian | -0.030 (-0.107-0.046) | 0.035 (-0.008-0.078) | 0.035 (-0.007-0.077) | 0.029 (-0.018-0.077) |
| Others | 0.009 (-0.033-0.051) | 0.009 (-0.049-0.067) | 0.010 (-0.048-0.069) | -0.016 (-0.057-0.025) |
| **Marital status** |  |  |  |  |
| Married | ref. | ref. | ref. | ref. |
| Widowed | 0.045 (-0.008-0.097) | -0.041 (-0.121-0.039) | -0.032 (-0.111-0.046) | -0.035 (-0.103-0.033) |
| Others | -0.062 (-0.147-0.023) | -0.112 (-0.206--0.018) | -0.091 (-0.181-0.000) | -0.080 (-0.154--0.006) |
| **Living arrangement** |  |  |  |  |
| Alone | ref. | ref. | ref. | ref. |
| With spouse | -0.030 (-0.094-0.034) | -0.026 (-0.110-0.058) | -0.016 (-0.100-0.068) | -0.021 (-0.096-0.054) |
| With children | -0.026 (-0.086-0.034) | -0.017 (-0.101-0.067) | -0.005 (-0.088-0.079) | -0.004 (-0.079-0.071) |
| With others | -0.010 (-0.049-0.029) | 0.017 (-0.028-0.061) | 0.016 (-0.030-0.061) | 0.012 (-0.031-0.055) |
| **Working status** |  |  |  |  |
| Working | ref. | ref. | ref. | ref. |
| Previously worked | 0.050 (0.029-0.072) | 0.134 (0.109-0.160) | 0.128 (0.104-0.153) | 0.067 (0.044-0.090) |
| Never worked | 0.037 (0.012-0.063) | 0.118 (0.080-0.156) | 0.115 (0.077-0.153) | 0.068 (0.032-0.104) |
| **Health Insurance** |  |  |  |  |
| No | ref. | ref. | ref. | ref. |
| Yes | 0.012 (-0.006-0.030) | 0.018 (-0.005-0.042) | 0.021 (-0.002-0.043) | 0.013 (-0.008-0.034) |
| **States/UTs** |  |  |  |  |
| Jammu and Kashmir | ref. | ref. | ref. | ref. |
| Himachal Pradesh | 0.073 (0.010-0.136) | -0.154 (-0.238--0.070) | -0.215 (-0.291--0.138) | -0.114 (-0.186--0.042) |
| Punjab | 0.151 (0.091-0.210) | -0.066 (-0.149-0.017) | -0.099 (-0.180--0.019) | -0.039 (-0.112-0.034) |
| Chandigarh | 0.035 (-0.031-0.101) | -0.128 (-0.225--0.030) | -0.134 (-0.229--0.040) | -0.003 (-0.091-0.085) |
| Uttarakhand | 0.004 (-0.060-0.067) | -0.222 (-0.311--0.132) | -0.271 (-0.359--0.183) | -0.080 (-0.165-0.004) |
| Haryana | 0.016 (-0.043-0.076) | -0.046 (-0.123-0.030) | -0.092 (-0.167--0.016) | 0.057 (-0.022-0.137) |
| Delhi | -0.019 (-0.076-0.037) | -0.149 (-0.233--0.064) | -0.181 (-0.261--0.101) | -0.056 (-0.131-0.019) |
| Rajasthan | -0.052 (-0.105-0.001) | -0.116 (-0.196--0.037) | -0.155 (-0.230--0.081) | 0.004 (-0.065-0.073) |
| Uttar Pradesh | -0.108 (-0.157--0.059) | -0.197 (-0.266--0.128) | -0.239 (-0.306--0.171) | -0.014 (-0.080-0.053) |
| Bihar | -0.052 (-0.101--0.003) | -0.149 (-0.227--0.071) | -0.180 (-0.253--0.106) | -0.012 (-0.089-0.066) |
| Arunachal Pradesh | 0.037 (-0.043-0.116) | -0.167 (-0.276--0.058) | -0.257 (-0.372--0.142) | -0.173 (-0.256--0.089) |
| Nagaland | 0.152 (0.028-0.276) | -0.380 (-0.463--0.298) | -0.410 (-0.496--0.324) | -0.275 (-0.345--0.204) |
| Manipur | -0.015 (-0.088-0.057) | -0.152 (-0.249--0.056) | -0.196 (-0.287--0.105) | -0.069 (-0.140-0.002) |
| Mizoram | -0.082 (-0.170-0.005) | -0.032 (-0.136-0.072) | -0.143 (-0.235--0.051) | -0.058 (-0.151-0.036) |
| Tripura | 0.017 (-0.042-0.075) | -0.115 (-0.206--0.025) | -0.154 (-0.240--0.068) | -0.029 (-0.110-0.051) |
| Meghalaya | 0.122 (0.023-0.221) | -0.125 (-0.254-0.005) | -0.134 (-0.262--0.006) | -0.056 (-0.156-0.045) |
| Assam | 0.025 (-0.031-0.080) | -0.096 (-0.170--0.021) | -0.140 (-0.211--0.068) | -0.070 (-0.143-0.004) |
| West Bengal | -0.014 (-0.063-0.035) | -0.116 (-0.188--0.043) | -0.137 (-0.207--0.066) | -0.064 (-0.131-0.004) |
| Jharkhand | -0.009 (-0.062-0.045) | -0.239 (-0.312--0.165) | -0.269 (-0.340--0.198) | -0.115 (-0.183--0.046) |
| Odisha | -0.041 (-0.096-0.014) | -0.204 (-0.280--0.128) | -0.216 (-0.289--0.142) | -0.039 (-0.111-0.034) |
| Chhattisgarh | 0.036 (-0.022-0.094) | -0.325 (-0.405--0.244) | -0.348 (-0.429--0.267) | -0.142 (-0.216--0.068) |
| Madhya Pradesh | -0.075 (-0.127--0.022) | -0.256 (-0.352--0.160) | -0.277 (-0.368--0.186) | -0.074 (-0.153-0.006) |
| Gujarat | -0.025 (-0.081-0.032) | -0.271 (-0.353--0.189) | -0.328 (-0.405--0.251) | -0.107 (-0.174--0.039) |
| Daman & Diu | -0.002 (-0.068-0.063) | -0.187 (-0.284--0.091) | -0.222 (-0.318--0.127) | -0.073 (-0.159-0.013) |
| Dadra & Nagar Haveli | -0.006 (-0.068-0.055) | -0.296 (-0.393--0.199) | -0.319 (-0.416--0.222) | -0.128 (-0.217--0.039) |
| Maharashtra | 0.038 (-0.016-0.092) | -0.198 (-0.269--0.128) | -0.203 (-0.272--0.134) | -0.031 (-0.101-0.038) |
| Andhra Pradesh | 0.113 (0.062-0.165) | -0.102 (-0.177--0.028) | -0.109 (-0.181--0.037) | -0.023 (-0.093-0.048) |
| Karnataka | 0.002 (-0.052-0.056) | -0.243 (-0.317--0.170) | -0.249 (-0.321--0.177) | -0.033 (-0.117-0.050) |
| Goa | 0.047 (-0.009-0.102) | -0.057 (-0.137-0.024) | -0.060 (-0.139-0.019) | 0.033 (-0.047-0.113) |
| Lakshadweep | 0.141 (0.061-0.222) | -0.187 (-0.274--0.099) | -0.204 (-0.289--0.119) | -0.133 (-0.220--0.046) |
| Kerala | 0.052 (-0.002-0.106) | -0.223 (-0.300--0.146) | -0.238 (-0.312--0.164) | -0.093 (-0.161--0.024) |
| Tamil Nadu | -0.019 (-0.070-0.032) | -0.178 (-0.254--0.103) | -0.199 (-0.273--0.125) | -0.054 (-0.124-0.017) |
| Puducherry | 0.003 (-0.056-0.062) | -0.132 (-0.219--0.045) | -0.131 (-0.216--0.046) | 0.038 (-0.048-0.123) |
| Andaman & Nicobar Islands | 0.223 (0.138-0.308) | -0.084 (-0.182-0.013) | -0.110 (-0.199--0.021) | -0.101 (-0.183--0.019) |
| Telangana | 0.044 (-0.009-0.097) | -0.089 (-0.166--0.012) | -0.099 (-0.175--0.024) | 0.021 (-0.051-0.094) |
