# Supplementary material for: Awareness, treatment, and control of hypertension in adults aged 45 years and over and their spouses in India: A nationally representative cross-sectional study
Source: PLoS Med. 2021 Aug 24;18(8):e1003740. doi: 10.1371/journal.pmed.1003740 (PMC8425529; doi:10.1371/journal.pmed.1003740)
Supplement: S13 Table — (DOCX) [file pmed.1003740.s020.docx]

**S13 Table. Comparison of estimates of hypertension prevalence by age and sex with those in Geldsetzer and colleagues**

|  | **This study, LASI 2017-18** | | | |  | **Geldsetzer et al. (2018),^1^ DLHS-4 & AHS 2012-14** | |
| --- | --- | --- | --- | --- | --- | --- | --- |
|  | **Hypertension identified from** | | | |  | **Hypertension identified from** | |
|  | **BP or treatment** | | **BP** | |  | **BP** | |
| Age group | **Female, % (95% CI)** | **Male, % (95% CI)** | **Female, % (95% CI)** | **Male, % (95% CI)** |  | **Female, % (95% CI)** | **Male, % (95% CI)** |
| 46-55 years | 35.5 (20.4-22.1) | 35.5 (20.4-22.1) | 24.0 (20.4-22.1) | 28.8 (26.4-31.3) |  | 32.7 (32.2-33.2) | 33.7 (33.1 - 34.3 ) |
| 56-65 years | 47.8 (19.1-22.0) | 42.9 (19.1-22.0) | 32.5 (19.1-22.0) | 31.2 (28.9-33.5) |  | 41.2 (40.6-41.9) | 39.0 (38.3 - 39.6) |
| >65 years | 58.7 (18.6-20.9) | 48.2 (18.6-20.9) | 40.4 (18.6-20.9) | 34.9 (33.2-36.7) |  | 48.6 (47.9-49.3) | 43.4 (42.7 - 44.2) |

Table shows estimates of prevalence of hypertension. Columns headed “BP or treatment” give estimates based on hypertension defined as in this study as systolic blood pressure (BP) ≥140 mm Hg or diastolic BP ≥90 mm Hg or self-report of ever having been diagnosed with hypertension/high BP and currently taking medication or under salt/diet restriction to control BP. Columns headed “BP” give estimates based on hypertension defined as in Geldsetzer et al. (2018) as systolic BP ≥140 mm Hg or diastolic BP ≥90 mm Hg^4^ Geldsetzer et al. (2018) estimates are obtained from eTable 2 in the Supplementary Material of that paper. DLHS-4 is District Level Health Survey-4. AHS is the second update of the Annual Health Surveys. Both of these surveys were conducted between 2012 and 2014. LASI is the Longitudinal Survey of India conducted 2017/18 used in this study. See next table (S14 Table) for group sizes.

^1^ Geldsetzer P, Manne-Goehler J, Theilmann M, Davies JI, Awasthi A, Vollmer S, et al. Diabetes and hypertension in India: a nationally representative study of 1.3 million adults. JAMA Internal Medicine. 2018 Mar 1;178(3):363-72.
