# Supplementary material for: Awareness, treatment, and control of hypertension in adults aged 45 years and over and their spouses in India: A nationally representative cross-sectional study
Source: PLoS Med. 2021 Aug 24;18(8):e1003740. doi: 10.1371/journal.pmed.1003740 (PMC8425529; doi:10.1371/journal.pmed.1003740)
Supplement: S14 Table — (DOCX) [file pmed.1003740.s021.docx]

**S14 Table. Numbers of participants by age and sex used to estimate prevalence rates presented in Supplementary Table S13**

| **Age group** | **This study, LASI 2017-18** | |  | **Geldsetzer et al. (2018), DLHS-4 & AHS 2012-14** | |
| --- | --- | --- | --- | --- | --- |
|  | **Female, N** | **Male, N** |  | **Female, N** | **Male, N** |
| 46-55 years | 11,597 | 9,700 |  | 114,018 | 98,447 |
| 56-65 years | 10,049 | 8,274 |  | 75,911 | 75,029 |
| >65 years | 7,915 | 8,005 |  | 48,056 | 54,197 |

Geldsetzer et al. (2018) numbers are from Table 1 in that paper
