# Supplementary material for: Awareness, treatment, and control of hypertension in adults aged 45 years and over and their spouses in India: A nationally representative cross-sectional study
Source: PLoS Med. 2021 Aug 24;18(8):e1003740. doi: 10.1371/journal.pmed.1003740 (PMC8425529; doi:10.1371/journal.pmed.1003740)
Supplement: Attachment — Submitted filename: Reply_text _R1_07072021.docx [file pmed.1003740.s023.docx]

Dear Editor,

Many thanks for giving us the opportunity to revise our paper. We are grateful for the detailed comments you and the reviewers provided. They have helped us to improve the clarity of the paper. This note provides our response to each of the comments.

**Requests from the editors:**

1. Please can the authors clarify their response ('No - some restrictions will apply) to the Data Availability question in the introductory form, as it appears that the data used are publicly available.

**Reply**: The data are indeed publicly available. We indicated that some restrictions apply in getting access because we cannot upload the data ourselves. Researchers must request access by completing this data request form <https://iipsindia.ac.in/sites/default/files/LASI_DataRequestForm_0.pdf> and sending it to datacenter@iipsindia.ac.in

2. Please add a completed STROBE/RECORD checklist as a supplementary file, labelled "S1_STROBE_Checklist" or similar and referred to as such in the Methods section.

In the checklist, please refer to individual items by section (e.g., "Methods") and paragraph number, not by line or page numbers as these generally change in the event of publication.

**Reply**: Added as S1 Checklist.

3. Please update the manuscript to include line numbering in the margins.

**Reply**: Done.

4. Please update the manuscript title such that the age range studied comes before the colon (e.g. Awareness, treatment, and control of hypertension in adults aged 45+ in India: a nationally representative cross-sectional study)

**Reply**: We have implemented this suggestion and we also added “and their spouses” as suggested by Reviewer 3. The revised title reads:

“Awareness, treatment, and control of hypertension in adults aged 45+ and their spouses in India: a nationally representative cross-sectional study”

5. Please add a new final sentence to the "Methods and findings" subsection of your abstract, which should begin "Study limitations include..." or similar and should quote 2-3 of the study's main limitations.

**Reply**: We have added the following sentence. “Study limitations included reliance in BP measurement on a single occasion, missing measurements of BP for some participants, and lack of data on non-adherence to medication.”

6. Please add basic sample demographic information to the abstract (median age and % male/female at a minimum).

**Reply**: We added this sentence at the beginning of the results section of the abstract: “The 64,427 participants in the analysis sample had a median age of 57 years, 58% were female and 70% were rural dwellers.”.

7. Please present conclusions in the past tense (e.g. in "Conclusion" section of abstract –

'Hypertension prevalence was high' rather than 'is high'. Please check throughout manuscript.

**Reply**: Done.

8. Please change the style of all in-text citations. These should be in square brackets and occur before punctuation.

**Reply**: Done.

9. Please remove the information on funding and data availability from the main text. In the event of publication, this information will appear in the article metadata via entries in the submission form.

**Reply**: Done.

10. Please remove the section 'Role of the funding source' from the manuscript.

**Reply**: Done.

11. Please add 1-2 sentences at the beginning of the discussion summarising why the study was performed followed by the major findings.

**Reply**: We added the following sentence.

“Even though, in India, hypertension is the main risk factor for CVD [10], accounts for the largest disease burden of any condition [3], and is substantially more prevalent among older adults [8], there were no nationally representative and state-specific estimates of hypertension awareness, treatment, and control for the older population in India.”

12. The discussion sentence introduces new data that is not described in the results section text (notably Spearman rank correlation rho and associated p values). Please ensure that any results discussed in the discussion section are first described adequately in the results section.

**Reply**: Done.

13. In the final paragraph of the discussion, please update the first sentence to include '...to our knowledge, this study produced the first estimates of hypertension ATC...'

**Reply**: Done.

14. Please ensure references do not contain bold or italicised text, and please note that references should list the first six authors followed by et al. where appropriate.

**Reply**: Done.

15. If an analysis plan was prepared prior to the study, please could this be provided in the supplementary materials.

Reply: We have now stated at the end of the Study design sub-section of the Methods section that we did not prespecify the statistical analysis. While we did not write a full analysis plan prior to undertaking the study, we did set objectives, select the data, define outcomes, identify many of the covariates, and describe many of the statistical methods in a brief 2-page document prior to examining the data. However, since this falls short of a full analysis plan and we did not release it before starting the data analysis, we judge that it would not be appropriate to include it in the Supplementary Material.

16. Please confirm that the maps used to produce Figure 3 and supplementary figure D2 are appropriate for publication under a Creative Commons CC-BY licence.

**Reply**: Yes, these figures are appropriate for publication under a Creative Commons CC-BY licence.

17. In tables that state P values, please update any P = 0.000 values to P < 0.001.

**Reply**: Done

**Comments from the reviewers:**

**Reviewer #1**: This is a cross-sectional analysis of hypertension "care cascades" using nationally representative data from older adults in India collected in the most recent LASI cohort wave. The study is well conducted, advances knowledge on the epidemiology of hypertension care in India, and is well-written in its framing relative to prior literature. I think the paper would be a good fit at PLOS Medicine but also could be improved through several suggested revisions.

1. The authors should provide clinical justification for why they have selected a BP threshold of <140/90 to define hypertension and hypertension control. I am not familiar with hypertension management guidelines in India, but this is a controversial topic and major guidelines including the ACC/AHA and WHO-PEN now recommend more strict thresholds in individuals with higher cardiovascular risk. Please see my comment below on limitations for more on my perspective on this issue.

**Reply**: We have now made clear in the Measures sub-section of the Methods section that the BP thresholds used to define both hypertension and hypertension control are those specified in the training module of the Indian National Programme for Prevention and Control of Cancer, Diabetes, Cardiovascular Diseases and Stroke (MoHFW, 2017), which seemed to us to be the most context appropriate definition for the purpose of our study. These official guidelines issued by the Government of India, unlike the other guidelines the reviewer refers to, do not recommend or require treatment for hypertension at BP< 140/90 in the presence of other CVD risk factors. The training module states that pharmacological and non-pharmacological treatments and advice should be offered at BP ≥ 140/90, and that the goal is to control BP below those thresholds (please see line number 151-164) .

2. I agree with primarily presenting sex- and age-adjusted estimates in the state-level data. However, the methods for age-sex adjustments for hypertension prevalence, awareness, treatment, and control is not 100% clear to me. Am I correctly inferring that the authors ran the logistic regression models with state fixed effects as well as fixed effects for sex + age groups (categorical rather than continuous age), and then used "average adjusted predictions" for each state?

**Reply**: Yes, this is what we did. We have clarified this in S4 Text and corrected an inaccuracy in the previous description of the method, which mistakenly referred to average marginal effects rather than average predictions.

3. The authors mention that they are enclosing their Stata.do files. This would be useful and welcome. I do not see the files in the submission, however, so I have been unable to personally review any statistical code.

**Reply**: We are sorry that you could not locate the Stata do file. It was available in the “other section” of the material related to the submission. You can find it at **S1 code**.

4. Figure 3: would strongly urge the others to redraw the heatmap using color gradients rather than color tertiles for proportions by state. Much granularity of information is lost using the tertiles.

**Reply**: Done. We agree that the figure is more informative this way.

5. This is a stylistic suggestion, but I **would recommend moving the table of odds ratios** (Table 4) to the appendix and instead present a table of average marginal effects from the logistic models. Such a table would be much easier for readers to interpret the absolute differences in proportions within categories of covariates. Additionally, in place of a table, I would favor using forestplots to present regression outputs visually; in Stata, "coefplot" is a very nice package to do this, if the authors would like to pursue this route.

**Reply:** Thanks for these suggestions. We have replaced odds ratios with averaged marginal effects, which we agree are more informative, and presented them in forestplots. Please see Figure 4.

5. I would request that the authors add a supplementary appendix specifying the precise survey questions that were used in LASI to define awareness and treatment. This can be a simple table and will help readers understand the numerators for the cascades as you define them.

**Reply**: Done. See S2 Text Outcome definitions.

7. Some states had higher proportions of individuals controlled than treated. I believe that this is because the authors have not defined the hypertension cascades to be conditional, i.e., achieving one step was not necessarily contingent on the prior step. For example, an individual could have reported a prior diagnosis of hypertension, not be receiving treatment, and yet still have a BP level under the controlled threshold. Such unconditional cascades are reasonable (and my own personal preference in these analyses), though I believe the Prenissl India study used conditional cascades. Since the finding of % control > % treated appears a bit unanticipated, especially to a reader less familiar with the definitional nuances of these studies, the authors may wish to add 1-2 lines in either a caption or main text describing why this finding can occur.

**Reply**: In response to this comment, the following one, and Reviewer #3 comment1, and Reviewer #4 comment 4, we have revised the definition of hypertension such that a participant reporting ever having been told that they had hypertension/high BP is not sufficient to classify the participant as hypertensive. We now require that those making such a report must also have reported either taking medication to control BP at the time of the survey or being under salt/diet restriction to control BP. Using this definition, the rate of control is lower than the rate of treatment in all states.

8. The authors have defined hypertension treatment strictly as pharmacological treatment. Why? Non-pharmacological therapy is effective for lowering blood pressure in clinical trials, and, LASI also had at least one question specifically on lifestyle changes for treatment of blood pressure (diet/salt focused). I believe the authors should either (1) specifically state why they opted to exclude non-pharmacological treatment in their estimates, (2) include diet as "treatment" in the main analysis, or (3) generate a sensitivity analysis in which diet treatment was also considered "treatment."

**Reply**: We agree that there is little or no justification to restrict the definition of treatment to pharmacological therapies. As explained in response to the previous comment, we have revised the definition of hypertension such that reported diagnosis must be accompanied by reported treatment/actions to control BP in order for the participant to be classified as hypertensive (unless they have measured BP above the hypertension thresholds). In the revised analysis, we defined treatment as reporting either currently taking medication to control BP or being under salt or diet restriction to control BP.

9. I wonder if a missing opportunity in this manuscript is at least exploring some state-level characteristics that are associated with hypertension prevalence and ATC. This need not be a complex formal analysis. For example, the authors could consider simply plotting proportions by state-level GDP or some other marker of economic development (as in the Prenissl paper). The authors comment on this already in the discussion (second paragraph, bottom of page 10).

**Reply**: Thank you for this suggestion. Figure 3 now shows scatter plots and regression best-fit lines for each outcome against Net State Domestic Product (NSDP) Per Capita, 2017 across states. It reveals positive relationships between all outcomes and NSDP per capita. We now comment on these relationships in the Results section and also draw attention to states that perform better and worse than would be expected on the basis of their NSDP (according to the linear model predictions) (see line number 296-305).

10. The supplementary tables E11 and E12 are very useful in helping readers locate this study in the context of the prior Geldsetzer paper in JAMA IM. Thanks for including it.

**Reply**: We are pleased that the tables are appreciated.

11. I can think of a few additional limitations that the authors may wish to mention:

* I agree that measurement error is probably the biggest limitation of this and other cross-sectional assessments of hypertension. I might just also add text that the hypertension prevalence is likely overestimated. This idea is suggested but not specifically stated.

**Reply**: In the paragraph on limitations in the discussion, we have now stated that it is likely that we overestimated prevalence (please see line numbers 457-461).

* Lower age limit of LASI <45 years cuts off a substantial absolute number of adults with hypertension at younger ages, given that India has a relatively young population age distribution.

**Reply**: We have now acknowledged this limitation in the paragraph referred to immediately above and referred to the Geldsetzer et al (2018) and Prenissl et al. (2019) studies for complementary evidence covering the younger population (please see line number 481-485).

* LASI does not assess if a respondent has ever had blood pressure measured (sometimes worded as "ever screened" in cascade papers). This is often a step in the hypertension cascade that is reported when available.

**Reply**. We have acknowledged this in the paragraph on limitations and conceded that it prevented us from both examining screening as a first step in the hypertension care cascade and distinguishing between the unaware who had never been screened and those unaware despite having had their BP measured (please see line number 457-459).

* I mentioned the issue of non-pharmacological therapy above. If the authors do not wish to address this in the analysis, then it should be mentioned as a limitation.

**Reply**: We have extended the definition of treatment to address this previous limitation.

* A conceptual limitation of the present study is that it focuses on the "treat-to-target" approach for hypertension treatment. As the authors may be aware, the field has been moving away from this approach to hypertension treatment, which is the one operationalized in this study. This shift has happened due to evidence that the relative benefits of hypertension therapy are constant across risk levels; thus, individuals with higher baseline risk have much larger absolute benefit irrespective of blood pressure. In other words, even a person with "normal" blood pressure according to traditional BP thresholds may have large benefit from BP therapy. This approach is sometimes referred to as "benefit-based" therapy and has begun to be incorporated into recent guidelines from the ACC/AHA and WHO-PEN.

**Reply:** We have now discussed this issue, very much along the lines suggested, in a new penultimate paragraph of the paper (please see line number 490-498).

**Reviewer #2:** I confine my remarks to statistical aspects of this paper. The general approach is fine, but I do have some questions and issues to resolve before I can recommend publication

[We have numbered the reviewer’s comments.]

1. NOTE: Line numbers would have made the review process easier.

**Reply**. Apologies. Line numbers have been added.

2. p. 4  In addition to "44% of global deaths ...." give a number of deaths, or rate per 100,000 or something.

**Reply**: We have added that there were 42 million deaths due to non-communicable diseases in 2019 (please see line number 96-97).

3. p. 5 While your results should definitely be useful, it IS possible to estimate these numbers using the analyses that have already been done. Those estimates might be poor, but, for instance, you could estimate the rates for older people in districts where they are not known by adjusting the rates in known districts using the more general results that were gotten in all districts.

**Reply**: We have revised the argument made in the third paragraph of the Introduction by adding the text shown below in italics.

“Estimates from a nationally representative sample of the population aged 18-49 [13], and from samples that included older adults but did not have nationwide coverage [14-20], do not permit evaluation of hypertension management across the country in the age group most prone to the condition and its related risks *without making strong modelling assumptions*.”

In the second paragraph of the Discussion, we have added:

“Previous estimates of ATC rates in the older Indian population that used data on these outcomes for older people in only six states [17] relied on the strong assumption that the patterns observed in those states would hold in other states. We showed substantial variation across states in rates of ATC and in the socioeconomic gradient of each outcome.” (please see line number 381-384).

We realize that the reviewer was probably referring to text a little further down in the Discussion, where we state:

“But that study could not estimate hypertension ATC, and the only previous nationally representative study of these outcomes was restricted to the 15-49 age range in which hypertension is much less prevalent [13].”

The first part of this statement is correct. The study referred to did not collect data on ATC outcomes. Modelling could indeed be done to extrapolate from states in which these outcomes are observed for both younger and older adults. However, that would rely on very strong assumptions. We believe that it is best not to return to this point again here because it would interrupt the flow of the comparison we make between our estimates and those obtained by other studies. And the interruption would be done only to say that something could be done but it would not deliver a very reliable estimate.

4. p. 6 It would be good to clarify that you can be a, b, and c .... or other combinations. It seems to me that you can be a) alone, a) and b), or a), b) and c).  But it would aid clarity to spell this out.

**Reply**: In response to comments from the other reviewers, we changed the definition of hypertension. We have revised the text in the Measures subsection of the Methods section accordingly. There is now only an a) and a b), with b) being different from the one defined before. We state that the participant was defined as having hypertension if they satisfied condition a) or condition b). This implies that they qualified if they satisfied a) and b). We refrain from using and/or since it is unnecessary. We have provided a precise definition of all outcomes in S2 Text.

5. p. 7 Why did you use the concentration index? What does it add to the regression results? It's a pretty unusual statistic, so there should be some reason for using it.

**Reply**: We have added that the concentration index allowed measurement of socioeconomic inequality along the full distribution of MPCE. And that it avoided loss of information from grouping participants and making comparisons only between extremes, e.g. richest fifth versus poorest fifth. The regression results are complementary in that they allowed examination of the association between each outcome and MPCE quintiles conditional on covariates. But they are more restrictive than the concentration index because participants are put into MPCE groups (quintiles), and the variation within those groups is lost (please see line number 191-196).

6. Why was BP unmeasurable on some people? Were these people different from those where it could be measured? Why wasn't multiple imputation used? (I think it should be, but you may have some reason for not doing so)

**Reply**: We have now stated the reasons BP could not be measured for some participants at the beginning of the Results (please see line number 208-212). Specifically, BP could not be measured if the cuff of the monitor could not be fitted to either of a participant’s arms because of problems like rash, cuts, edema, eczema, open sores, wounds, significant bruise. Also, BP was not measured if a participant did not give consent and, obviously, it was not measured in proxy interviews. We have referred to a new S1 Table that compares sociodemographic characteristics of participants with and without BP measurements and noted differences in the text. Those for whom BP could not be measured tended to be richer, better educated, and older, and they were more likely to be urban, a privileged caste, Muslim, and not working. In the limitations part of the Discussion section (please see line number 464-475), we have discussed the potential biases arising from these systematic differences, which are statistically significant but not substantial.

While multiple imputation is indeed very useful in limiting excessive loss of data, addressing potential bias and increasing precision in the presence of substantial item non-response, we opted for complete case analysis/listwise deletion as appropriate the method for handling missing values in view of the relatively low rate of missing BP measurements of just 9% A large-scale general-purpose population survey of older adults, which had a baseline analysis sample of 64,427 participants even after dropping those with incomplete item response, allowed us to obtain estimates with a relatively high degree of precision. While this strategy may come at the cost of some bias in terms of estimated overall prevalence rates, we do not believe that the use of multiple imputation would substantially alter the socio-demographic patterns documented in the paper. We do not expect large correlations of the socio-demographics examined with variables external to our model that may influence BP. Moreover, the potential benefits of multiple imputation relative to complete case analysis/listwise deletion in our application need to be balanced with its potential costs in terms of additional modelling assumptions implied by the imputation model, such as variable selection, distributional assumptions, and estimation method. These methodological choices could have implications of potential misclassifications resulting from imputed BP for the entire ATC cascade, which unfolds at the level of an individual observation and thus crucially relies on the accurate classification of individual observations. Reproducibility of results derived from imputations (the current public release of the LASI file does not include “official” imputations for missing BP measurements generated by the data producers) would be another issue. Yet another would be the correct inference procedures for the analyses, which may require substantial adjustments to some estimation procedures used in our study, such as the estimation of concentration indices. In view of these trade-offs, we preferred to stick to complete case analysis/listwise deletion, also since this procedure still appears to be the most widely accepted form of missing handling values.

7. Figure 3: It would be better to use more colors and to have the percent relate to some quality of the color (e.g. saturation). A very good site on doing this well is ColorBrewer [https://colorbrewer2.org/#type=sequential&scheme=BuGn&n=3](https://eur03.safelinks.protection.outlook.com/?url=https%3A%2F%2Fcolorbrewer2.org%2F%23type%3Dsequential%26scheme%3DBuGn%26n%3D3&data=04%7C01%7Codonnell%40ese.eur.nl%7Cce4f0dfd8556465892ac08d93159f91d%7C715902d6f63e4b8d929b4bb170bad492%7C0%7C0%7C637595084795877806%7CUnknown%7CTWFpbGZsb3d8eyJWIjoiMC4wLjAwMDAiLCJQIjoiV2luMzIiLCJBTiI6Ik1haWwiLCJXVCI6Mn0%3D%7C1000&sdata=WeIzAEzGRlUraRzyI7YRODKmlNPHpvSXeUsFmdHrGos%3D&reserved=0)

**Reply**: Thanks for the suggestion. We have refined the colours of all the maps.

**Reviewer #3:** The authors provide a clear picture of hypertension prevalence and rates of awareness, treatment, and control among older adults in India. The research idea is a valuable extension of existing evidence on younger age groups and highlights the need for better hypertension care among the population segment most at risk of suffering from hypertension and cardiovascular diseases.

[We have numbered the reviewer’s comments.]

1. However, the definition of their sample needs to be improved. The authors defined participants who have "ever […] been diagnosed as hypertensive" (p. 6) to have hypertension. The survey instrument does not distinguish between hypertension and high blood pressure. Thus, this is a rather broad indicator, which does not reflect clinical guidelines and can capture temporary episodes of high blood pressure. I assume that the inclusion of "having been diagnosed" in the study sample is the reason why in some age groups and states the control rates are larger than the treatment rates (Table E3, Bihar, for example). The authors should base the definition of hypertension on BP and self-reported medication use only.

**Reply**: We have revised the definition of hypertension along the lines suggested. Specifically, hypertension is now defined as BP≥140/90 or self-reported currently taking medication or under salt/diet restriction to control BP. See the revised Measurements subsection of the Methods section (line number 151-164) and the new S2 Text that gives precise definitions of all outcomes. We also include non-pharmacological treatment (salt/diet restriction) in the definition of hypertension/hypertension treatment in response to Reviewer #1, comment 8. Using the new definition, the estimated rate of hypertension control was lower than the rate of treatment for all sociodemographic groups and all states.

Additional specific comments:

2. Last sentence in first paragraph of introduction (p.4) is too long

**Reply**: We have rewritten the text and split it into two sentences.

3. Average of BP measures (p.6.): were there missing values in either the second or the third measurement? If yes, it needs **to be stated** how the final BP was computed if only one of the two measurements was available.

**Reply**: We have clarified in the Measurements subsection of the Methods section: data shows that “All three readings of BP was available for 65,751 participants and for remaining participants (6,449) no reading was available”. Data does not shows any such cases with single or only two readings

4. MPCE indicator (p. 6, "Measures"): It is not clear to me why all expenditure on healthcare and medicine were excluded from the calculation. This should briefly be explained.

**Reply**: As explained in S3 Text, we excluded expenditure on healthcare and medicine to avoid such expenditures, which may well be temporary and not financed from a household’s current income, inflating total household expenditure and giving a false impression of a higher standard of living. We have now given a concise version of this justification in the main text (Measurement subsection of Methods section) (please see line number 166-171)..

5. Levels of Covariates (p.6, "Statistical Analysis"): The main text, appendix or notes of Table 1 should include an overview on how levels of categories were chosen and which information they include. Eg. what categories were clubbed in the "other" groups, how were years of education cut-offs defined. Furthermore, it should be considered if the categories in "other" really are comparable or should better be listed separately, despite their small sample size.

**Reply**: We have added an explanation of the year of schooling categorisation in the main text (Statistical analysis, first paragraph) and listed the sub-groups within each of the Other categories in the notes to Table 1.

6. Missingness in variables of interest (p. 7, "Results"): Roughly 10% of observations have either a missing value in the BP measurement or another variable. The appendix should include a comparison of the sample characteristics of the samples with and without missing in the variables of interest (BP measurement, ATC variables). A detailed table of the number (%) of missing values in each outcome and covariate should be provided.

**Reply**: Please see S1 Table for comparisons between participants with and without BP measurements, and our response to Reviewer #2 comment 6 regarding missing on BP measurement. We have added S2 Table that gives the number and percent missing on each variable and referred to this table in the main text at the beginning of the Results section. We have added S3 Table that compares estimates of hypertension prevalence and rates of ATC obtained from the full item response analysis sample and from a sample that does not drop participants with missing information on MPCE or any sociodemographic. We refer this table also at the beginning of the Results section.

7. Median age (p.8): IQR should be reported alongside the median.

**Reply**: Done.

8. Thresholds for comparison (p.9 "Figure 2 shows […] in Haryana"): The threshold for comparison, eg "the rate of awareness exceeded 70% in only three states…" seem arbitrary and are confusing. I suggest deleting these kinds of comparisons.

**Reply**: Done.

9. Figure 3: The thresholds for the colour coding vary in each panel. Either, the same cut-offs or different colours in each panel should be used. Or it should be made clearer that the focus is on the relative comparison across states, e.g. by removing the prevalence ranges from the legend. The same applies to the other maps of this kind.

**Reply**: For rates of awareness and treatment, we have now used the same scale/thresholds. For control, we use a different scale because the rates are much lower, and the map would show very little variation in control rates if we used the same scale as used for awareness and treatment. This is consistent with the approach taken in the Prenissl et al (2019) paper published in PLOS Medicine. We reverse the colouring for hypertension prevalence (because higher is worse in this case) and allow the scale to differ from that used for ATC because prevalence is estimated from a different sample from that used for ATC. We believe that it is preferable to show the numerical scales in order that readers get relevant information.

10. Multivariate regression (p. 10): I assume the authors mean "multivariable logistic regression"

**Reply**: We have changed the term to multivariable logistic regression.

11. Presentation of results in Discussion section: The authors present new results in the discussion. The discussion should only briefly summarize the main results (without presenting p-values and CIs). The focus should be on the interpretation of the results and putting them into context.

**Reply**: The Discussion has been revised as suggested.

12. Comparison to previous evidence from other studies in Discussion: This is interesting but too detailed. A higher level comparison would be more suitable.

**Reply**: We have reduced the level of detail in this discussion.

13. In several instances, the authors refer to the poverty rates across states. It would be interesting to include this information in figures/tables; eg. by colour coding Figure 4 according to poverty rate quartile.

**Reply**: In response to this comment and Reviewer #1 comment 9 we have added Figure 3 that shows scatter plots and regression best-fit lines for each outcome against Net State Domestic Product (NSDP) Per Capita, 2017 across states. It reveals positive relationships between all outcomes and NSDP per capita. We now comment on these relationships in the Results section and place less emphasis on the associations with poverty rates.

14. Decimal places: The authors should report only two decimal places, also when presenting the concentration index results.

**Reply**: We have reported to no more than two decimal places for all results except for concentration index estimates and P-values. We prefer to report concentration indices to three decimal places because small values of this index can still reflect a good deal of inequality. For example, there is a 15 percentage point difference in hypertension prevalence between the richest and poorest MPCE quintile groups, but the respective concentration index is only 0.061. Reporting concentration indices to only two decimal places would introduce a level of inaccuracy much greater, in relative terms, than that arising from reporting other estimates to the same number of decimal places.

15. Oxford comma: The Oxford comma should be used consistently.

Reply. Done.

**Reviewer #4:**

1.    The authors used some acronyms in the abstract, which can be avoided if not well-known ones like ATC.

**Reply**: We no longer use ATC in the abstract. We continue to use BP for blood pressure because this is a sufficiently well-known abbreviation.

2.About 10% of participants were excluded from the study due to some reasons. There may be a possibility that the excluded participants may have different socioeconomic characteristics. Authors may check that the participants included in the study are not different from excluded participants.

**Reply**: Please see S1 Table for comparisons between participants with and without BP measurements, and our response to Reviewer #2 comment 6 regarding missing on BP measurement. We have added S2 Table that gives the number and percent missing on each variable and referred to this table in the main text at the beginning of the Results section. We have added S3 Table that compares estimates of hypertension prevalence and rates of ATC obtained from the full item response analysis sample and from a sample that does not drop participants with missing information on MPCE or any sociodemographic. We refer this table also at the beginning of the Results section (please see line number 208-219).

3. The initial recommendation of hypertension control is lifestyle modification. If this information is available, authors may think about modifying treatment definition in the cascade of care.

**Reply**: We agree that there is little or no justification to restrict the definition of treatment to pharmacological therapies. We have redefined treatment as reporting either currently taking medication to control BP or under salt or diet restriction to control BP.

4. "STATA"	is a typo. Please have a look:  [https://www.stata.com/statalist/archive/2010-01/msg00489.html](https://eur03.safelinks.protection.outlook.com/?url=https%3A%2F%2Fwww.stata.com%2Fstatalist%2Farchive%2F2010-01%2Fmsg00489.html&data=04%7C01%7Codonnell%40ese.eur.nl%7Cce4f0dfd8556465892ac08d93159f91d%7C715902d6f63e4b8d929b4bb170bad492%7C0%7C0%7C637595084795877806%7CUnknown%7CTWFpbGZsb3d8eyJWIjoiMC4wLjAwMDAiLCJQIjoiV2luMzIiLCJBTiI6Ik1haWwiLCJXVCI6Mn0%3D%7C1000&sdata=1V2V7lFOE55Rpi7Z0UbQ8lF3eAjASi59DYUa2Xl6KzY%3D&reserved=0)

**Reply**: We have used Stata.

5, Authors mentioned, "Table 1 shows characteristics of the full analysis sample and estimates of age-sex adjusted hypertension prevalence. The median age of the sample was 57 years." The measure of central tendency gives only a half picture. Please use a measure of dispersion along with a measure of central tendency here and throughout the manuscript.

**Reply**: We have provided the interquartile range in addition to the median age.

6. The study's title, all the tables, and figure captions indicate that analysis is only for 45+ aged participants, but about 9% of participants were <45 years as per table 1. Please make it clear.

**Reply**: In the Sample design subsection of the Methods section we stated that the sample includes individuals aged 45+ and their spouses. We now state at the beginning of the Statistical analysis subsection that we used data on individuals aged 45+ and their spouses who could be younger than 45. We have revised the title to make clear that the study delivered evidence for older adults aged 45+ and spouses: “Awareness, treatment, and control of hypertension in adults aged 45+ and their spouses in India: a nationally representative cross-sectional study.

7. The authors used Spearman rank correlation to measure the relationship between hypertension prevalence, awareness, and treatment. Some other methods may be preferred in such cases.

**Reply**: In the revised text, we cite correlation only between concentration indices of the different outcomes across states. We prefer a rank correlation statistic since we are interested in whether states that rank higher with respect to inequality in hypertension prevalence also rank higher on inequality in rates of ATC. We have confirmed that the associations are robust to using Pearson correlation.

8. Figure 2 and figure 3 seems duplication of information; authors may choose one of them.

**Reply**: We have dropped what was Figure 2, retained what was Figure 3 (maps) – now Fig 2 – and, following a suggestion of Reviewer #1, added scatter plots of the state-specific outcomes against state net domestic product – now Fig. 3.

9. In Figure 3, the authors categorized the prevalence of hypertension prevalence and cascade of care, masking the state-level variation. It may be worth to show it using a continuous scale.

**Reply**: We have replaced the previous maps with heat maps that show more variation.

10. Authors mentioned, "Table 4 shows estimated OR from a multivariate logistic regression for each outcome." Although it is used as a synonym, there is a difference in multivariable and multivariate regression. Authors may recheck that they used multivariate or multivariable regression.

**Reply**: We have used now used the term multivariable regression.

11. In table 4, the authors categorized age in various age groups, which can lead to loss of information and an increase in type I and II errors. A supplementary analysis is warranted in the case of categorization of continuous data.

**Reply**: We categorized age to allow for nonlinearities that would be missed if the linear correlation with continuous age were examined or if continuous age was entered linearly in the regressions. Fitting low order polynomials in age would be more restrictive and higher order polynomials would be difficult to interpret and present easily. It is very common in descriptive epidemiological analyses published in medical journals of the sort we present to show variation in outcomes over age groups.

12. The authors included caste and religion as independent variables in table 4, but biologically caste and religion have nothing to do with hypertension and its cascade of care. Authors may think about removing caste and religion or may be interested in assessing the impact of all other variables excluding caste and religion in a separate analysis.

**Reply**: We are not examining associations that are determined by biology. Awareness, treatment, and control of hypertension depend on information, access to healthcare, income, health behaviour, and other factors that are determined by socioeconomic circumstances, as well as demographic characteristics. Onset of hypertension itself highly dependent on sociodemographic factors. Caste and religion and religion are included because each reflects and is related to socioeconomic circumstances. This is most obvious for caste. But socioeconomic conditions, as well as culture and diet that can impact on hypertension and its management, also differ across religions in India. Hence, we strongly prefer to retain caste and religion in the analysis. In any case, it is only in the multivariable regression analysis that their retention impacts on the (conditional) associations found between the outcomes and other factors.

13. Authors mentioned, "The discrepancies are partly explained by the facts that we examined a much older age range and found, like other studies." Discrepancy may not be the correct word choice as the differences are obvious with higher age, especially in postmenopausal women.

**Reply**: We have replaced “discrepancies” with “differences”.

**References**

Ministry of Health and Family Welfare (MoHFW). Training Module for Medical Officers for Prevention, Control and Population Level Screening of Hypertension, Diabetes and Common Cancer (Oral, Breast & Cervical). National Programme for Prevention and Control of Cancer, Diabetes, Cardiovascular Diseases and Stroke. New Delhi: Government of India, 2017. <https://main.mohfw.gov.in/sites/default/files/Training%20Module%20for%20Medical%20Officers%20for%20Prevention%2C%20Control%20and%20Population%20Level%20Screening%20of%20NCDs_1.pdf> (Accessed June 29, 2021).
