# Supplementary material for: Awareness, treatment, and control of hypertension in adults aged 45 years and over and their spouses in India: A nationally representative cross-sectional study
Source: PLoS Med. 2021 Aug 24;18(8):e1003740. doi: 10.1371/journal.pmed.1003740 (PMC8425529; doi:10.1371/journal.pmed.1003740)
Supplement: Attachment — Submitted filename: ReplyR2_190721.docx [file pmed.1003740.s024.docx]

**20.07.21**

**Point by point Reply to Editor**

1. Please update ‘aged 45+’ to ‘aged 45 years and over’ throughout the manuscript (including in the title).

Reply: Done

2. Thank you for responding to my previous query regarding your data availability statement, it appears that your data sharing approach aligns with the journal policy. While you have clarified the matter for me, please can you update your response to read ‘No – some restrictions will apply’ as it did in the first revision, as this is more accurate.

Reply: Done

3. In the ‘Describe where data can be found’ section of the submission form:

* you state ‘Data is publicly available’ – please update to ‘Data are publicly available’. Please update elsewhere in the manuscript if necessary to ensure data are treated as plural.

* Please add a sentence to the effect of ‘data will be made available to the researchers meeting the criteria for access to confidential data’, if applicable and accurate.

Reply: Done

4. In both the abstract and discussion, please update the sentence ‘Study limitations included reliance in BP’ to ‘Study limitations included reliance on BP’.

Reply: Done

5. Please provide 95% CIs and p values for quantitative results presented in the abstract, as appropriate.

Reply: Done

6. Please consider whether any quantitative data ought to be added to the abstract to support the results detailed on lines 29-32. If you opt to include, please ensure it is presented with 95% CIs and p-values, where appropriate.

Reply: We have added estimates of differences between groups, along with 95% CIs and p-values.

7. The first sentence of the ‘Notes’ under Table 1 (‘Adjusted for age and sex, except age (sex) group estimates adjusted for sex (age) composition’) is confusing, please reword to improve clarity.

Reply: Done (also for notes to Table 3).

8. In several tables and figures, you need to define the abbreviation MPCE in the legend. Please check and update throughout.

Reply: Done

9. Table 3 still contains p-values of 0.000. Please update these to P<0.001.

Reply: Done

10. Please ensure consistent use of ‘Figure’ (as opposed to ‘Fig’).

Reply: Done

11. On lines 328-329, please report the actual p-values rather than stating p-value<0.05.

Reply: Done

12. The first line of your discussion should be split into two sentences. I would advise placing a full stop after [8] on line 362, then beginning the following sentence ‘Yet, to our knowledge, there were no nationally representative…’ etc.

Reply: Done

13. Please add the date accessed for references 24 and 31.

Reply: Done

14. Please review your references and ensure that initials are not followed by a full stop (as in, for example, reference 32), and also please ensure journal titles are abbreviated consistently (e.g. reference 33 should be ‘Circ Res’.

Reply: Done

Comments from Reviewers:

Reviewer #1: I have reviewed the author responses in detail. My concerns have been very thoughtfully addressed, and I have no further critiques.

I wish to commend the authors for making their full statistical code available and for generally completing a very thoughtful revision.

This is a very nicely done paper and the authors should be proud.

Reply: Thank you so much for your kind words

Reviewer #2: The authors have addressed my concerns and I now recommend publication.

Reply: Thank you so much

Reviewer #4: As authors have addressed almost all of my comments, I recommend the acceptance of the paper.

Reply: Thank you so much
